# Evolution of *UCP1* Gene and Its Significance to Temperature Adaptation in Rodents

**DOI:** 10.3390/ijms26052155

**Published:** 2025-02-27

**Authors:** Xinyue Liang, Minyu Wu, Qiuting Nong, Siqi Yang, Tuo Kan, Ping Feng

**Affiliations:** 1 Key Laboratory of Ecology of Rare and Endangered Species and Environmental Protection, Ministry of Education of the People’s Republic of China, Guangxi Normal University, Guilin 541006, China; 2 Guangxi Key Laboratory of Rare and Endangered Animal Ecology, Guangxi Normal University, Guilin 541006, China

**Keywords:** *UCP1* gene, evolution, ambient temperature, rodent

## Abstract

Adaptive thermogenesis comprises shivering thermogenesis dependent on skeletal muscles and non-shivering thermogenesis (NST) mediated by uncoupling protein 1 (UCP1). Although the thermogenic function of *UCP1* was adopted early in some placental mammals, positive selection predominantly occurred in the ancestral branches of small-bodied species. Some previous studies have revealed that rodents living in northern or high mountain regions adapt to cold environments by increasing NST, whereas those living in tropical and subtropical regions that are not exposed to cold stress express low concentrations of *UCP1*, indicating that *UCP1* may have evolved to adapt to ambient temperatures. In this study, we explored the evolution of *UCP1* and its significance to temperature adaptation by performing detailed evolutionary and statistical analyses on 64 rodents with known genomes. As a result, a total of 71 *UCP1* gene sequences were obtained, including 47 intact genes, 22 partial genes, and 2 pseudogenes. Further, 47 intact genes and 3 previously published intact *UCP1* genes were incorporated into evolutionary analyses, and correlation analyses between evolutionary rate and ambient temperatures (including average annual temperature, maximum temperature, and minimum temperature) of the rodent survives were conducted. The results show that *UCP1* is under purifying selection (*ω* = 0.11), and among rodents with intact *UCP1* sequences, *Urocitellus parryii* and *Dicrostonyx groenlandicus*—the two species with the lowest ambient temperatures among the rodents used here—have higher evolutionary rates than others. In the statistical analyses, in addition to ambient temperatures, body weight and weight at birth were also taken into account since weight was previously proposed to be linked to *UCP1* evolution. The results showed that after controlling for the phylogenetic effect, the maximum temperature was significantly negatively correlated with the evolutionary rate of *UCP1*, whereas weight did not have a relationship with *UCP1* evolutionary rate. Consequently, it is suggested that ambient temperature can drive the evolution of rodent *UCP1*, thereby enhancing NST adaptation to cold stress.

## 1. Introduction

Adaptive thermogenesis is generally divided into shivering thermogenesis (ST), which depends on skeletal muscles, and non-shivering thermogenesis (NST), which relies on brown adipose tissue (BAT) [1]. BAT takes charge of producing heat under cold stress through NST, and it is unique to mammals [2,3]. This tissue is rich in small-bodied species and neonates of large-bodied species [4], and it plays an essential role in maintaining high body temperature [2,5] or in facilitating rewarming after hibernation [1], which enables them to exploit cold environments. In some mammals (e.g., rodents), NST increasingly functions as a substitution for shivering thermogenesis when they are repeatedly subject to cold stress [6]. Uncoupling protein 1 (UCP1), a carrier protein with cold-inducible expression, is highly expressed in the mitochondria of BAT, and it governs the mechanism of NST by boosting the proton leakage in mitochondria, releasing the oxidation energy into heat [2].

The evolutionary rate of *UCP1* in mammals is greater than that of other vertebrates (fish and amphibians) [7,8]. Some studies investigated the selective pressure imposed on eutherian branches with intact *UCP1* sequences and found that *UCP1* was under strong selection and coincided with keeping the adaptive function [5], agreeing with the suggestion that *UCP1* plays a fundamental role in dealing with cold stress in eutherians [9,10,11]. Chronic cold stress stimulates BAT proliferation and activity [12]. Notably, although placental mammals recruited *UCP1* for the thermogenic function in an early period, positive selection signatures are detected in the ancestral branches of both Glires (rodents and lagomorphs) and Afroinsectivores, respectively, indicating the critical role of UCP1 in these groups on thermogenesis [7]. Some studies investigated the thermogenesis of small-sized mammals and found that rodents living in northern or high mountain regions adapted to cold stress by increasing NST [13], whereas rodents living in tropical and subtropical regions that are not subjected to cold stress showed a low concentration of UCP1 in their mitochondria of BAT [6,13]. These results suggest that *UCP1* may have evolved to respond to the changes of ambient temperature.

Uncoupling protein 1 can function in thermogenesis induced by cold or diet and body temperature maintenance, and it also has a significant impact on metabolism and energy balance [14,15,16]. The link between *UCP1* evolution and body temperature, *UCP1* evolution and body weight varies across species. For example, Gagnon et al. [17] explored the genetic variation of *UCP1* in the population of savannah monkeys and found that the majority of derived allele frequencies correlated with solar irradiance but not overall low temperatures. In horses, it was reported that serum UCP1 concentration had no relationship with body weight [18]. However, in adult goats and growing kids, a negative correlation was suggested between rectal temperature and UCP1 concentration [16,18]. UCP1 and its orthologs not only exist in placental mammals but it is also identified in ectothermic animals [19]. In the common carp (*Cyprinus carpio*), the concentration of UCP1 is rich in the liver and less so in the kidneys [20]. Although the expression of UCP1 in murine brown fat was up-regulated when exposed to cold conditions [21,22], the expression of common carp hepatic UCP1 was lower at 8 °C when compared with those acclimated at 20 °C [23]. Similar results were detected in gilthead sea bream (*Sparus aurata*), with a lower level of hepatic expression in winter than in summer and autumn [20,24]. The distinct responses of UCP1 between mammals and ectothermic animals during cold exposure may be attributed to the different expressed tissues of UCP1 in these two kinds of animals. In mammals, UCP1 is highly expressed in brown adipocytes, but in carp, UCP1 is not found in adipose tissues, which indicates the potentially functional discrepancy between placental mammals and fish [20,23].

Uncoupling protein 1 is mainly expressed in small-sized mammals [25], and the dependence of some mammals on UCP1 for heat production may have begun to decrease or diminish as body weight increased [9,26]. For example, *UCP1* merely momentarily appeared in the BAT of neonatal cattle [27] and pre-weaned harp seals (*Pagophilus groenlandicus*) [28], and it is also reported that in some larger-bodied animals (such as humans), BAT is present in neonates and disappears in adults [25]. This is also supported by the suggestion that body size is inversely related to NST capacity [1] and that *UCP1* in eutherian mammals with higher body weight at birth tended to be pseudogenized [29]. In addition, in marine mammals, the pseudogenization of *UCP1* is presumed to link with large body size (such as in elephant seals) [29,30], and lifestyle can affect *UCP1* evolution to some extent, with *UCP1* in most semiaquatic species (e.g., the harbor seal and Antarctic fur seal) being under strong selective pressure while being lost in fully aquatic mammals [29].

In sum, *UCP1* evolution may primarily be driven by ambient temperature and body size. In previous studies, Gaudry et al. [5] demonstrated that the inactivation of *UCP1* was a historical contingency in some mammals and among the 141 vertebrates used 22 rodents were included; meanwhile, Mendes et al. [7] investigated the evolution of *UCP1* in 252 vertebrates (19 were rodents) and found that the evolutionary rate of *UCP1* was increased in mammalians when compared with that of other *UCP*s. Of the rodents sampled in these two studies, 18 species are overlapped, and the sample size is small. At present, with the advances in sequencing technology, more and more genome data are accumulated, which provides the opportunity to investigate the evolution of *UCP1* in rodents. In addition, data on ambient temperatures and body weight are convenient to obtain. Thus, exploring the relationship between the evolutionary rate of *UCP1* and ambient temperature in rodents can be realized; and since it is suggested that *UCP1*-dependent thermogenesis may decrease with increasing body size, we will also examine the relationship between evolutionary rate of *UCP1* and body weight. Therefore, in this study, we selected rodents as the research objects, and the aims are 1. to investigate the evolution of *UCP1* and 2. to explore whether ambient temperature and body weight are related to the evolutionary rate of *UCP1*, by collecting rodent genomes and their related ambient temperatures and body weights as much as possible.

## 2. Results

### 2.1. Data Collection

**Figure 1:**
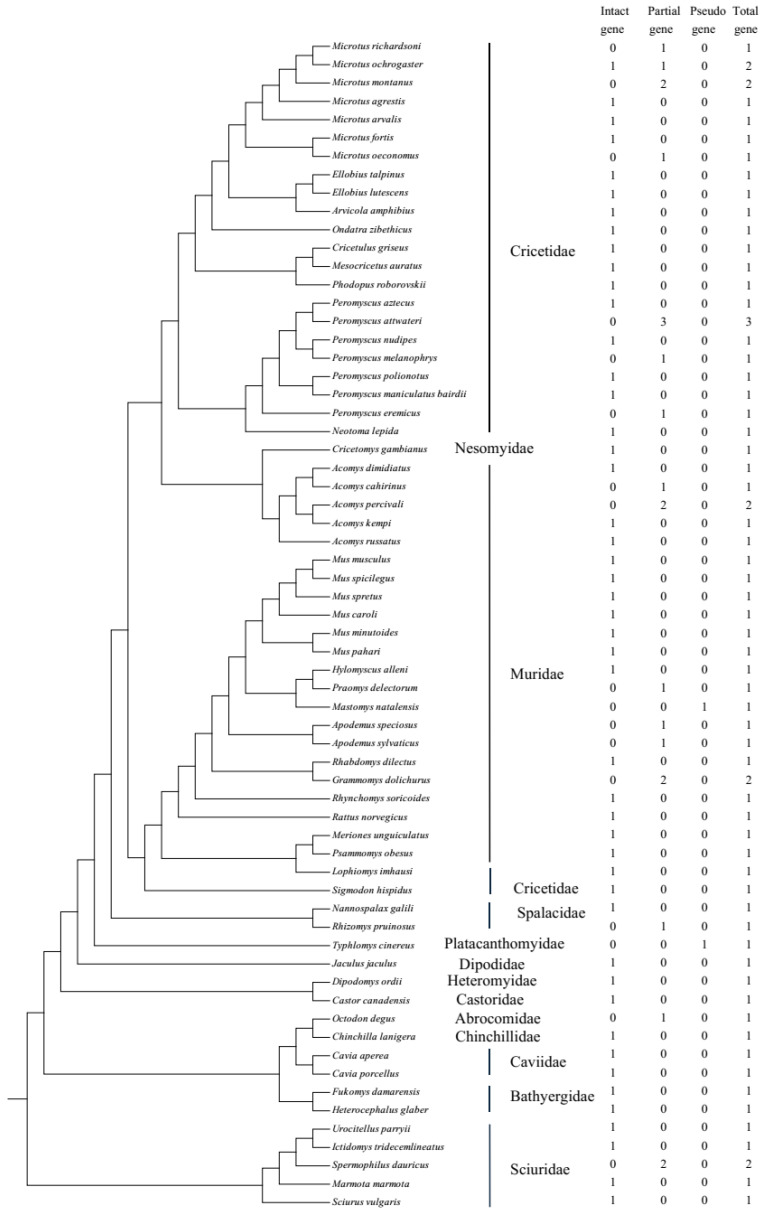
The distribution of the *UCP1* gene in rodents used in this study.

Sixty-four genomes of rodents from 13 families were downloaded, with the genome coverage from 1× to 8356.0× (two species had no genome coverage data) and contig N50 from 1 kb to 122.6 Mb (please see Table S1). After searching against 64 publicly available rodent genomes, 71 *UCP1* gene sequences were obtained, including 47 intact genes, 22 partial genes, and 2 pseudogenes, and this distribution is displayed in Figure 1, with the phylogenetic tree referring to Alvarez-Carretero et al. [31]. The *UCP1* gene obtained from rodents consists of six exons with a total length of 924 bp, and “Ns” (i.e., unsequenced nucleotide) in exon6 of *Microtus oeconomus UCP1* was detected, which is due to either incomplete genome sequencing or poor genomic assembly in this region, whereas in *Jaculus jaculus*, a 3 bp insertion exists in exon1. In general, a rodent species has only one intact *UCP1*, if any; some species have more than one partial gene because gene fragments are identified in different scaffolds (Table S2). For example, in *Grammomys dolichurus*, exon5/6 and exon2/3 are from two scaffolds, respectively; thus, they are assumed to originate from two genes. In *Microtus montanus* and *Acomys percivali*, exon1/2/5/6 and exon3/4 are from two scaffolds, while in *Apodemus sylvaticus*, exon1/2/6 and exon3/4/5 are from two scaffolds; considering that these exons of the three species may be mismatched on different scaffolds, we tried to concatenate them by the order exon1-exon6 and found that they were either intact genes or pseudogenes, and it was hard to judge whether they were correct or not. Thus, we still viewed them as partial genes. As for the pseudogenes, *Mastomys natalensis* and *Typhlomys cinereus* each had one pseudogene, with 1 bp deletion in exon2 and 1 bp insertion in exon5, respectively. In addition, three intact *UCP1* sequences (accession Nos. AF515781.1, AF271263.1, and MT114186.1) were downloaded from NCBI and then added into the intact gene mentioned above; thus, in total, 50 intact *UCP1* genes were obtained and would be applied to the following phylogenetic tree reconstruction and selective analyses. All the intact sequences used in this study are listed in Supplementary Text S1.

**Figure 2:**
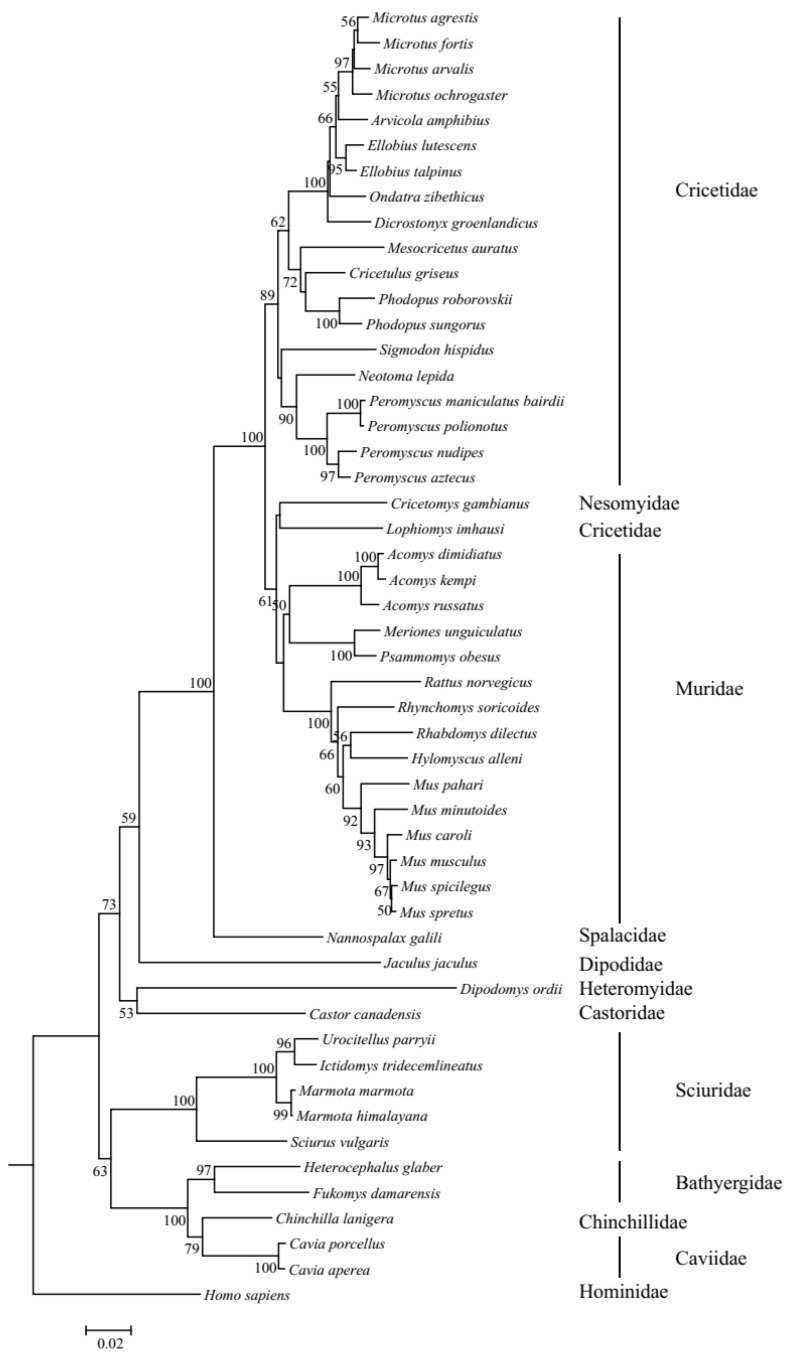
NJ tree of *UCP1* genes in rodents. Bootstrap value < 50 is not shown.

The resulting phylogenetic tree of *UCP1* genes revealed that the ML tree (Figure S1) and NJ tree (Figure 2) were inconsistent in several species. Specifically, in the ML tree, *Lophiomys imhausi*, a species of Cricetidae, was mixed with species of the Muridae group; meanwhile, in the NJ tree, *Lophiomys imhausi* clustered with *Cricetomys gambianus* to form a sister group to the Muridae group. In addition, the position of the Sciuridae group is also different between the ML tree and NJ tree. Due to the fact that the bootstrap values of the NJ tree are higher than those of the ML tree, we used the NJ tree as the guide tree for the following selective analyses. The topology of the NJ tree is different from the tree shown in Figure 1, especially in the inner topology of the Cricetidae group, and this difference may be caused by incomplete lineage sorting [32,33]. The ambient temperatures that rodents survive vary frequently; thus, we collected them as average temperature, maximum temperature, and minimum temperature by primarily referring to Alhajeri et al. [34]; in brief, average temperature means average annual temperature and maximum temperature and minimum temperature refer to the highest temperature of the warmest month and the lowest temperature of the coldest month, respectively. As for the species that have no ambient temperature in Alhajeri et al. [34], *Cavia porcellus*’s temperature data were gathered from Animal Diversity Web (https://animaldiversity.org (accessed on 5 May 2023)), while *Nannospalax galili*’s data were collected from Sumbera et al. [35], though the average temperatures were absent in them. Body weights were mainly collected from Alhajeri et al. [34], in AnAge: The Animal Ageing and Longevity Database (https://genomics.senescence.info/species/index.html (accessed on 5 May 2023)), and sometimes from Animal Diversity Web and other sources (Table S3). Weights at birth were also collected, and they were from AnAge: The Animal Ageing and Longevity Database. In total, the weight at birth of 32 species was gathered. The data and references for temperature, body weight, and weight at birth are listed in Table S3.

### 2.2. Selective Pressure Analyses on UCP1 Gene

The branch analyses implemented in Paml4 software allow the generation of different *ω* values for distinct branches in a given phylogenetic tree. The acquisition of BAT-mediated NST is related to the rodent *UCP1* gene, which promotes the occurrence of futile cycles in the BAT mitochondria and the release of metabolic heat [36,37]. Hence, through the comparison of the obtained *ω* values, it is possible to evaluate the selective forces operating along different branches of rodents. In the selective analyses, the results showed that the *ω* value for Model0 (one ratio) was 0.11, and the comparison between Model0 (one ratio) and Model0 (*ω* = 1) suggested that Model0 (one ratio) fitted the dataset better (*p* < 0.05), indicating that the *UCP1* gene was subject to purifying selection in rodents. In addition, comparative analyses of the three pairs of site models M1a vs. M2a, M7 vs. M8, and M8 vs. M8a revealed that no positively selected sites existed in *UCP1* genes. Results are summarized in Table 1.

In our free-ratio analyses, the results showed that the *UCP1* sequences of most species were subject to strong purifying selection, with *ω* being small (Table S3). However, it is necessary to point out that the *ω* of *UCP1* in *Acomys dimidiatus* was 999, which is due to the zero synonymous substitution rate in this gene; thus, *ω* of *Acomys dimidiatus UCP1* was discarded in the subsequent statistical analyses. *D*_N_*/d*s for *Urocitellus parryii*, *Cavia porcellus*, and *Peromyscus maniculatus bairdii* was 0.61, 0.44, and 0.39, respectively, larger than that of many of the species used in this study. Considering that these species inhabit cold environments or occupy a wide range of thermal niches, we wonder whether the *ω* of the *UCP1* gene is correlated with the ambient temperature of the habitat. It is worth mentioning that, among the species surveyed here, *Urocitellus parryii* and *Dicrostonyx groenlandicus* can survive under extreme cold conditions, which is different from other rodents. Therefore, to investigate whether the evolution of the *Urocitellus parryii* and *Dicrostonyx groenlandicus UCP1* gene sequences is different from that of other species, a two-ratio model (Model A) analysis was conducted by assigning the branches leading to these species as the foreground branch with the rest as the background branch. The results showed that *UCP1* of *Urocitellus parryii* and *Dicrostonyx groenlandicus* exhibited a nearly two-fold increment in the *ω* value in comparison with other rodents’ counterparts (*ω*_1_ = 0.28 vs. *ω*_2_ = 0.11, *p* < 0.05), suggesting an accelerated evolution of the *UCP1* gene in *Urocitellus parryii* and *Dicrostonyx groenlandicus*. A further test was carried out to examine whether the foreground branches were under relaxed selection or not using Relax (www.datamonkey.org/RELAX (accessed on 3 June 2023)) [38], and the test found that selection relaxation (K = 0.47) was significant (*p* = 0.044, LR = 4.04).

### 2.3. Correlation Between Evolutionary Rate and Temperature, Evolutionary Rate and Body Weight

**Figure 3:**
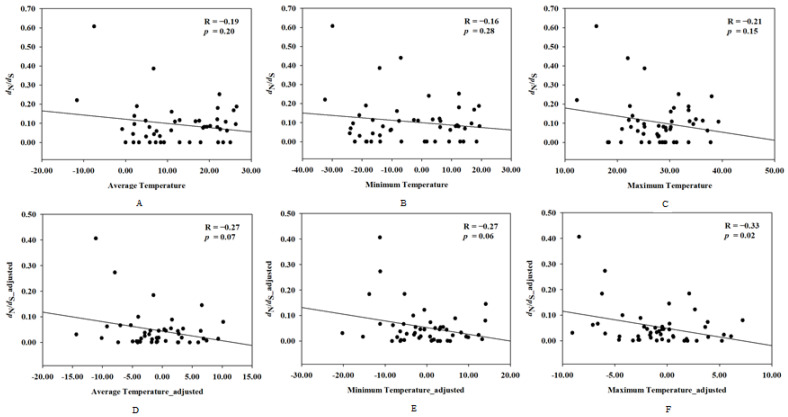
The correlation between the evolutionary rate of *UCP1* and ambient temperature before and after controlling for phylogenetic inertia. *X*-axis denotes *d*_N_/*d*_S_ (*ω*) and *d*_N_/*d*_S__adjusted, whereas *Y*-axis indicates (**A**) average temperature; (**B**) minimum temperature; (**C**) maximum temperature; (**D**) average temperature_adjusted; (**E**) minimum temperature_adjusted; (**F**) maximum temperature_adjusted.

To investigate the correlation between the evolutionary rate of *UCP1* and the rodents’ ambient temperature, correlation analyses were performed between the evolutionary rate of *UCP1* and the mean temperature, maximum temperature, and minimum temperature, respectively, and the results showed that the evolutionary rate of *UCP1* has no relationship with the ambient temperature in rodents (for average temperature: R = −0.19, *p* = 0.20; for minimum temperature: R = −0.16, *p* = 0.28; for maximum temperature: R = −0.21, *p* = 0.15); however, after controlling for the phylogenetic effect, the maximum temperature was negatively correlated to evolutionary rate of *UCP1* (R = −0.33, *p* = 0.02), whereas no significant correlation existed between the average temperature and the evolutionary rate of *UCP1* (R = −0.27, *p* = 0.07) or the minimum temperature and the evolutionary rate of *UCP1* (R = −0.27, *p* = 0.06) (Figure 3).

Body weight data were primarily collected from Alhajeri et al. [34], and when the data were absent, we searched for them on AnAge: The Animal Ageing and Longevity Database. Meanwhile, we also collected the data of weight at birth for the species used in this study as much as possible. In total, 50 species had both *UCP1* evolutionary rates and body weight data, and among them, the body weight of 33 and 17 species were collected from Alhajeri et al. [34] and AnAge: The Animal Ageing and Longevity Database, respectively. After removing *Acomys dimidiatus* due to a *ω* = 999 of *UCP1*, 49 species were used in the analysis. In addition, 32 species had weight at birth data, and they are from AnAge: The Animal Ageing and Longevity Database. Correlation analyses were conducted between body mass and evolutionary rate and weight at birth and evolutionary rate, and the results suggested that no significant association was found (Table S4), no matter whether controlling for the phylogenetic signal or not.

## 3. Discussion

In mammals, non-shivering thermogenesis mediated by UCP1 is a critical mechanism for coping with cold exposure [7,39]. In this study, we employed the recent release of rodent genomes to explore the evolution of *UCP1* and its relationship to habitat temperature and body weight. After the investigation of the *UCP1* gene distribution in rodents, we found that 17 of the 64 rodent species did not have any intact *UCP1* genes, of which 15 species each had at least one partial gene whereas 2 species each had one pseudogene. Two pseudogenes were from *Mastomys natalensis* (1 bp deletion in exon2) and *Typhlomys cinereus* (1 bp insertion in exon5). The indels that were not a multiple of 3 resulted in an altered open reading frame (ORF) and premature stop codons, which are hallmarks of pseudogenes [40]. Considering that the mean temperature of the habitats of *Mastomys natalensis* and *Typhlomys cinereus* was 24.1 °C and 16.6 °C, respectively, the minimum temperature of the habitats of both species was above 2 °C [34], and UCP1 was mainly expressed to deal with cold pressure, we inferred that although they did not have an intact *UCP1* gene, the ambient temperatures were relatively high and the disruption of *UCP1* did not have a major impact on their lives.

Selective pressure analysis of 50 species with intact *UCP1* genes showed that the overall selection pressure on the *UCP1* gene in rodent species was estimated to be 0.11, suggesting that the gene was under purifying selection, which is in line with two previous studies using species from several taxonomic groups [7,41]. The two-ratio model showed that the *ω* values of foreground branches consisting of *Urocitellus parryii* and *Dicrostonyx groenlandicus*, respectively, which survived under extreme cold conditions, were significantly higher than those of the background branches of other rodents. This suggested that the evolution of the *UCP1* genes of *Urocitellus parryii* and *Dicrostonyx groenlandicus* were under accelerated evolution compared with that of other rodents, which may allow more sensitivity to the mutations responsive to the environment. On the other hand, a higher *UCP1* evolutionary rate may also appear to experience a relaxed constraint, which allows for more sensitivity to mutations resulting in neofunctionalization [7], followed by purifying selection, just as *UCP1* is assumed to be relaxed in animals with large body weights due to its possible enhancement in muscle thermogenesis, such as in elephant seals [29]

Several factors were suggested to affect the energy expenditure of small-sized animals, including phylogeny, body size, and climate, of which microclimate and body size seem to be the most important [13]. In general, climate conditions have an impact on the temperature difference between mammal bodies and the environment [42]. UCP1-mediated NST, induced by the cold, is an essential manner of heat production in small mammals, and it is viewed as an important mechanism in contributing to the survival of animals in the cold [13]. NST varies with season and temperature, it is generally high in winter or cold [43], and the abilities of the thermogenesis of small-sized animals change according to the habitat environment [44]. In this study, we performed a linear regression analysis using *ω* and habitat temperature, and the results showed that after removing the phylogenetic effect, *ω* was negatively correlated with the maximum habitat temperature, indicating that selection pressure on *UCP1* increased with decreasing habitat temperature. The significant correlation between *ω* and habitat temperature also demonstrated that habitat temperature is an important factor driving the evolution of *UCP1*. The results showed that the evolutionary rate of *UCP1* is negatively correlated with the maximum temperature in rodents used here; that is, when the evolutionary rate is higher, the maximum temperature will be lower. This is consistent with the plateau pika (*Ochotona curzoniae*), a species that maintains body temperature through augmenting NST in response to the cold stress in the Qinghai–Tibet Plateau [13]. Also, species living in warm environments may lose UCP1 function due to the disappearance of selective pressure acting on NST [4]; in addition, the emergence of adaptive behavior such as building nests to protect the young from cold exposure can attenuate the burden of thermogenesis, compensating for *UCP1* pseudogenization [2].

*Urocitellus parryii* mainly lives in high altitudes in the Arctic and tundra regions [45,46], where its habitat has a minimum temperature of −29.9 °C and a mean temperature of −7.5 °C [34]. The results in the two-ratio model (*ω*_1_ = 0.28, *ω*_2_ = 0.11, *p* = 0.04) also indicated that there was a faster evolutionary rate of the *UCP1* gene in *Urocitellus parryii* and *Dicrostonyx groenlandicus* compared with other rodents and also suggested that habitat temperature may be an important factor driving the evolution of *UCP1*. In addition, *Cavia porcellus* is distributed in a large scope of elevations, ranging from sea level to 4000 m [47], and it can survive in a wide range of temperatures, from 22 °C to −7 °C, though it is suggested that it cannot tolerate extreme temperatures [48,49,50]. *Peromyscus maniculatus bairdii* has a wide distribution and is common in grasslands, scrublands, and woodlands [51,52], and the temperature range of its habitat is also wide, from 25.2 °C to −14.1 °C [34]. These two samples suggest that wide thermal niches may also drive the evolution of the *UCP1* gene.

This study also examines the correlation between body weight and the evolutionary rate of *UCP1*, due to previous suggestions that the NST is inversely correlated to body weight [1] and that when the body weight is above 10 kg, it has no contribution to heat production [9]. The results of this study demonstrated that although NST has a correlation with body weight in some species, this relationship cannot be applied to the rodent group. In addition, data on weight at birth were also collected to test a previous conclusion stating that mammals with a UCP1 pseudogene have a greater weight at birth [29]. Despite the fact that a positive selection acted on the lineage leading to small-sized species, emphasizing its importance at the early stage of this lineage, not all the small-sized species used NST mediated by UCP1, and the partial genes or pseudogenes in some rodents may due to other NST strategies that link to body size, behavioral adaptation, or the warm habitat they inhabit, and such manners reduce the reliance on UCP1 and further lead to the loss or pseudogenization of *UCP1* [7]. Furthermore, during the process of body weight collection, we found that the value of body weight varies among different sources in some species. Although we selected the data from the same source as much as possible, a single source is hard to cover all the body weight data of the rodents used here, and data from different sources will raise the problem of data inconsistency due to the criteria used in various sources. Thus, the relationship between body weight and evolutionary rate is roughly estimated. More data from the same source are expected in the future to further verify the result.

At last, for most small-sized species, the strategy of increasing heat production via NST to ensure a thermostatic life during chronic cold stress is particularly important [6], and higher UCP1 activity increases oxidative capacity, which is central to thermogenic processes [6], suggestive of the critical role on *UCP1* gene. This study only explored the primary influencing factors on *UCP1* evolution, and the conclusion that *UCP1* evolutionary rate correlated with maximum temperature but not body weight enhances our understanding of the evolution of *UCP1* in small-sized mammals. Other factors that have a driving force on the *UCP1* evolution still need to be further explored and verified.

## 4. Materials and Methods

### 4.1. Data Sources

Genome assemblies of rodent species were downloaded from the National Center for Biotechnology Information (NCBI, https://www.ncbi.nlm.nih.gov/, accessed on 13 October 2022), and the published *UCP1* sequences of rodents were also collected by searching NCBI and other sources. Information on ambient temperatures and body weight was collected from the Animal Diversity Web (https://animaldiversity.org/ (accessed on 5 May 2023)) and previously published literature [34]. Of note, body weight means adult weight, and during the data collection, weight at birth was also gathered when it was displayed in the above-mentioned sources.

### 4.2. Identification of UCP1 Gene

We used the published rodent *UCP1* gene (accession No. AF515781.1) as the reference sequence and performed TblastN to search for *UCP1* in rodents with known genome sequences. To identify all six exons that make up a *UCP1* gene, we downloaded the specific genomic scaffold containing *UCP1* and conducted Blast 2 between each exon from the reference sequence and the scaffold. Blast hit sequences were extended to both 5′ and 3′ directions along the genome scaffolds to attempt to identify the entire coding regions [40]. Subsequently, all 6 exons were assembled and compared with published *UCP1* data from closely related species using ClustalX1.83 [53], and indels (insertions/deletions) were recorded during the process. Newly identified *UCP1* sequences were classified into 3 categories: intact genes, partial genes, and pseudogenes. Intact genes refer to those that have full-length coding sequences and a proper start and stop codon; partial genes are those with a truncated open reading frame as a result of incomplete genome sequencing; and pseudogenes mean those with inactivated mutations [54].

### 4.3. Sequence Alignment and Phylogenetic Reconstruction

The resulting sequences were aligned with MEGA 6 [55] and checked by eye. The alignments of nucleotide sequencing were obtained according to protein sequence alignments and were subsequently used for selective pressure analyses. The phylogenetic tree of the *UCP1* gene was reconstructed by both Neighbor-Joining (NJ) [56] and Maximum Likelihood (ML) approaches supplemented in MEGA 6, with a *Homo sapiens UCP1* gene (accession No. NM_021833.5) as the outgroup gene. The NJ tree was reconstructed using the following settings: the Kimura 2-parameter model [57] was selected, the gaps/missing data were treated by complete deletion, and the number of bootstrap replicates was set to 1000 [58].

### 4.4. Selection Analyses

In order to investigate selective pressures acting on rodent *UCP1*, we estimated the ratio (*ω*) of the rate of nonsynonymous substitutions (*d*_N_) to the rate of synonymous substitutions (*d*_S_) using Paml4.7 [59]. When *ω* = 1, this means the gene is under neutral evolution; *ω* < 1 indicates negative or purifying selection whereas *ω* > 1 denotes positive selection. M0 (one ratio) assumes that all branches in the evolutionary tree have the same *ω* ratio; however, in M0 (*ω* = 1), the *ω* value is fixed to 1 [59]. A two-ratio model that allows the *ω* value to vary between the background and foreground branches was employed and then compared with M0 (one ratio). This approach was used to compare the evolutionary rates of *UCP1* between specific rodent species and the rest using the likelihood ratio test (LRT), which uses twice the log likelihood difference (2 dL) and parameter numbers difference of the two models as degree freedom to calculate the *p*-value with a chi-square distribution [7,60]. Additionally, a free-ratio model allowing all branches to have different *ω* values was conducted.

In addition, site model analyses were also conducted to detect positively selected sites. In this section, three pair model (M1a-M2a, M7-M8, and M8a-M8) analyses were carried out and compared. Of these, M1a/M7/M8a is a null/neutral model whereas M2a/M8 an alternative/positive selection model [61]. Similar to the branch model analyses mentioned above, a likelihood ratio test (LRT) was also applied to determine which model was more appropriate to the data. A significant difference in LRT (*p* < 0.05) denotes a positive selection model fits the dataset better than the neutral model. For the dataset that displays a significant difference in the LRT, the Bayes Empirical Bayes (BEB) method [62] supplemented in PAML software was executed to detect potentially positive selection signatures in the sites, and only the sites with posterior probability (PP) > 0.95 were considered.

### 4.5. Statistical Analysis

We explored the relationship between the *ω* of the *UCP1* gene and ambient temperature in rodents using correlation analysis in SPSS 27.0 (SPSS Inc., Chicago, IL, USA). Phylogenetic inertia must be considered during phylogenetic comparative analyses because closely related species are more likely to share similar characteristics. This shared inheritance can lead to data non-independence. To control for the effects of phylogenetic inertia, we additionally used phylogenetically independent contrasts [63], which were estimated by PDAP (Phenotypic Diversity Analysis Program, version 6.0) [64] implemented in Mesquite 2.74 [65] to transform the data before the correlation analyses. We used the species tree from Alvarez-Carretero et al. [31] as the guide tree. We also investigated the correlation between *UCP1* evolutionary rate and body weight using the same method.

## 5. Conclusions

This study investigated the evolution of *UCP1* and its related driving force in rodents. The results revealed that purifying selection governed the evolution of *UCP1* and that ambient temperature can drive the evolution of *UCP1*, particularly in species that inhabit cold regions, whereas body weight has no effect on *UCP1* evolution in rodents, which is probably due to their smaller sizes.

## Figures and Tables

**Table 1:** Selective pressure analyses of *UCP1* gene in rodents.

Model	Parameter Estimates	-LnL	2 dL (*p*-Value)	Positively Selected Sites
Model 0 (one ratio)	*ω* = 0.11	8727.64	M0 (one ratio) vs. M0 (*ω* = 1) 1647.74 (**0**)	None
Model 0 (*ω* = 1)	*ω* = 1.00	9551.51		
Model A (two ratios)	*ω*_1_ = 0.28, *ω*_2_ = 0.11	8725.50	Model A (two ratios) vs. M0(one ratio) 4.28 (**0.04**)	None
Model 1a (nearly neutral)	*p*_0_ = 0.87, *ω*_0_ = 0.05, *p*_1_ = 0.13, *ω*_1_ = 1.00	8588.74	M2a vs. M1a 0 (1)	None
Model 2a (positive selection)	*p*_0_ = 0.87, *ω*_0_ = 0.05, *p*_1_ = 0.13, *ω*_1_ = 1.00, *p*_2_ = 0.00, *ω*_2_ = 36.47	8588.74		
Model 7 (β)	*p* = 0.23, *q* = 1.73	8538.86	M8 vs. M7 0 (1)	None
Model 8 (β and *ω*)	*p*_0_ = 1.00, *p* = 0.23, *q* = 1.72, (*p*_1_ = 0) *ω* = 1.27	8538.86		
Model 8a (β and *ω* = 1)	*p*_0_ = 1.00, *p* = 0.23, *q* = 1.65, (*p*_1_ = 0) *ω* = 1.00	8539.11	M8 vs. M8a 0.50 (0.48)	None

Note: *p*-values < 0.05 are indicated in bold.

## Data Availability

Data are available in the Supplementary Materials.

## Supplementary Materials

The following supporting information can be downloaded at https://www.mdpi.com/article/10.3390/ijms26052155/s1. References [34,35,66,67,68] are cited in the Supplementary Materials.
