# Supplementary material for: Evolution of UCP1 Gene and Its Significance to Temperature Adaptation in Rodents
Source: Int J Mol Sci. 2025 Feb 27;26(5):2155. doi: 10.3390/ijms26052155 (PMC11899873; doi:10.3390/ijms26052155)
Supplement: Supplementary file 1 [file ijms-26-02155-s001.zip › ijms-3334673-supplementary.pdf]

Figure S1 ML tree of *UCP1* gene. Boostrap value<50 was not showed.

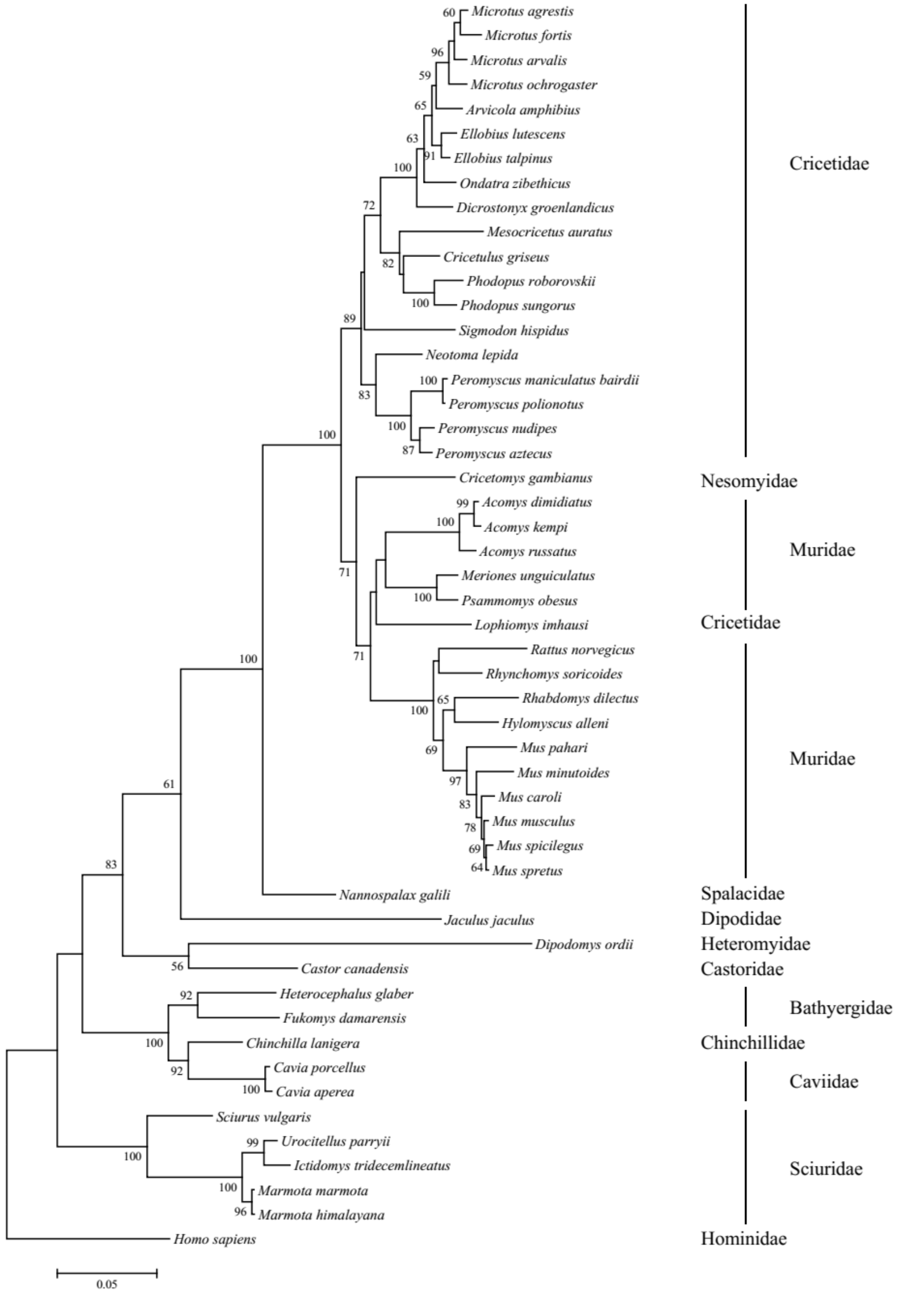

Table S1 Genome information of rodents (n=64).

Family	Species	Assembly ID	Assembly accession	Contig N50 (kb)	Genome coverage (x)
Abrocomidae	Octodon degus	375158	GCA_000260255.1	19.8	80.0
Bathyergidae	Fukomys damarensis	6721021	GCA_012274545.1	44.8	29.0
	Heterocephalus glaber	13382641	GCA_944319725.1	2900.0	55.0
Castoridae	Castor canadensis	5430091	GCA_009822645.1	86.9	87.0
Caviidae	Cavia aperea	177331	GCA_000688575.1	1.0	333.0
	Cavia porcellus	304568	GCA_000151735.1	80.6	6.8
Chinchillidae	Chinchilla lanigera	397218	GCA_000276665.1	61.1	87.0
Cricetidae	Arvicola amphibius	10049691	GCA_903992535.2	5400.0	45.0
	Cricetulus griseus	7269151	GCA_003668045.2	2700.0	31.0
	Ellobius lutescens	769091	GCA_001685075.1	11.6	130.0
	Ellobius talpinus	769101	GCA_001685095.1	8.3	50.0
	Mesocricetus auratus	12641341	GCA_023213145.1	25300.0	100.0
	Microtus agrestis	7175071	GCA_902806755.1	55.0	50.0
	Microtus arvalis	4065171	GCA_007455615.1	34.8	77.0
	Microtus fortis	13483651	GCA_014885135.2	90.7	108.0
	Microtus montanus	11145301	GCA_020392405.1	2.9	13.0
	Microtus ochrogaster	10918221	GCA_020004485.1	44.7	108.0
	Microtus oeconomus	4064071	GCA_007455595.1	51.2	77.0
	Microtus richardsoni	11143181	GCA_020387435.1	38.6	47.0
	Neotoma lepida	762981	GCA_001675575.1	9.6	48.0
	Ondatra zibethicus	2211371	GCA_004026605.1	73.7	43.9
	Peromyscus attwateri	4416691	GCA_902168425.1	20.5	50.0
	Peromyscus aztecus	4416631	GCA_902168405.1	19.2	50.0

	Peromyscus eremicus	5378821	GCA_902702925.1	64.3	50.0
	Peromyscus maniculatus bairdii	8338201	GCA_003704035.3	30.1	115.0
	Peromyscus melanophrys	4416661	GCA_902168415.1	15.8	50.0
	Peromyscus nudipes	4416581	GCA_902168325.1	19.8	50.0
	Peromyscus polionotus	2639781	GCA_003704135.2	13.2	290.0
	Phodopus roborovskii	13231611	GCA_943737965.1	25800.0	34.0
	Sigmodon hispidus	2210711	GCA_004025045.1	68.0	43.2
Dipodidae	Jaculus jaculus	11377851	GCA_020740685.1	22100.0	61.0
Heteromyidae	Dipodomys ordii	234081	GCA_000151885.2	48.1	-
Muridae	Acomys cahirinus	2211791	GCA_004027535.1	42.5	32.7
	Acomys dimidiatus	10452981	GCA_907164435.1	33.0	80.0
	Acomys kempi	10453051	GCA_907164505.1	33.6	85.0
	Acomys percivali	10453411	GCA_907169655.1	26.5	97.0
	Acomys russatus	7946671	GCA_903995435.1	4500.0	58.0
	Apodemus speciosus	1216231	GCA_002335545.1	4.0	65.0
	Apodemus sylvaticus	498341	GCA_001305905.1	1.2	17.0
	Grammomys dolichurus	10823141	GCA_019843835.1	5.4	26.3
	Hylomyscus alleni	10823151	GCA_019843855.1	4.2	31.2
	Mastomys natalensis	11852551	GCA_021653895.1	1100.0	53.0
	Meriones unguiculatus	4444301	GCA_008131255.1	58.7	1.0
	Mus caroli	16218941	GCA_900094665.2	122600.0	60.0
	Mus minutoides	9527551	GCA_902729485.2	103.7	80.0
	Mus musculus	7358741	GCA_000001635.9	106100.0	-
	Mus pahari	1086041	GCA_900095145.2	29.5	74.0
	Mus spicilegus	1861121	GCA_003336285.1	30.9	50.0

	Mus spretus	731341	GCA_001624865.1	17.9	60.0
	Praomys delectorum	10823131	GCA_019843815.1	13.9	26.3
	Psammomys obesus	10230921	GCA_907164565.1	77.2	85.0
	Rattus norvegicus	12837471	GCA_023515805.1	27800.0	28.0
	Rhabdomys dilectus	10824041	GCA_019844195.1	27.3	55.8
	Rhynchomys soricoides	10824011	GCA_019843965.1	79.8	56.4
Cricetidae	Lophiomys imhausi	10230911	GCA_907164525.1	35.8	82.0
Nesomyidae	Cricetomys gambianus	2211821	GCA_004027575.1	80.8	36.7
Platacanthomyidae	Typhlomys cinereus	12588411	GCA_023101885.1	2000.0	256.0
Sciuridae	Ictidomys tridecemlineatus	9297671	GCA_016881025.1	44.1	8356.0
	Marmota marmota	590591	GCA_001458135.1	66.5	30.0
	Sciurus vulgaris	7920861	GCA_902686455.2	16300.0	23.0
	Spermophilus dauricus	1262911	GCA_002406435.1	34.8	132.3
	Urocitellus parryii	1905031	GCA_003426925.1	91.0	40.0
Spalacidae	Nannospalax galili	182301	GCA_000622305.1	30.4	86.0
	Rhizomys pruinosus	5430111	GCA_009823505.1	2200.0	205.0

Note: “-” means no data.

Table S2 The detailed description of partial gene and pseudogene.

Species	Intact gene	Partial gene	Description on partial gene	Pseudogene	indels	Total gene
Octodon degus	0	1		0		1
Microtus ochrogaster	1	1	exon2-6	0		2
Microtus montanus	0	2	Exon1/2/5/6 and exon3/4 are from two scaffolds, respectively	0		2

Microtus oeconomus	0	1	“Ns” exist in exon6	0		1
Microtus richardsoni	0	1		0		1
Peromyscus attwateri	0	3	Exon1-2(a), exon1-2(b), and exon5-6 are from three scaffolds, respectively	0		3
Peromyscus eremicus	0	1		0		1
Peromyscus melanophrys	0	1		0		1
Acomys cahirinus	0	1		0		1
Acomys percivali	0	2	Exon3/4 and exon1/2/5/6 are from two scaffolds, respectively	0		2
Apodemus speciosus	0	1		0		1
Apodemus sylvaticus	0	2	Exon1/2/6 and exon3/4/5 are from two scaffolds, respectively	0		1
Grammomys dolichurus	0	2	Exon5/6 and exon2/3 are from two scaffolds, respectively	0		2
Mastomys natalensis	0	0		1	1bp deletion in exon2	1
Praomys delectorum	0	1		0		1
Typhlomys cinereus	0	0		1	1bp insertion in exon5	1
Spermophilus dauricus	0	2	Exon3/4 and exon1/2 are from two scaffolds, respectively	0		2
Rhizomys pruinosus	0	1		0		1

Table S3 Habitat temperature, body weight, weight at birth, related references, and the results of free ratio (evolutionary rate).

Species (n=50)	Average_T (℃)	Max_T (℃)	Min_T (℃)	Reference	Evolutionary rate	Body weight (g)	Reference	Weight at birth (g)	Reference
Fukomys damarensis	21.9	35	6	Alhajeri et al. (2020)	0.1204	81.95	Thomas et al (2016)	ND	ND
Heterocephalus glaber	25.8	33.6	17.6		0.1676	35	AnAge: The Animal Ageing and Longevity Database	2	AnAge: The Animal Ageing and Longevity Database
Castor canadensis	4.8	23.9	-16.4		0.1136	18750	Alhajeri et al. (2020)	430	
Cavia aperea	22	30.8	12.5		0.1797	637		59.66	
Cavia porcellus	ND	22	-7	Animal Diversity Web (https://animaldiversity.org)	0.4399	728	AnAge: The Animal Ageing and Longevity Database	85	
Chinchilla lanigera	12.9	22.2	3.7	Alhajeri et al. (2020)	0.1164	642.5		35	
Arvicola amphibius	2.7	22.4	-18.7		0.1895	200	Alhajeri et al. 2020	5	
Ellobius lutescens	10.9	29.3	-10.2		0.0628	70.4		ND	ND
Ellobius talpinus	7.2	29	-16.9		0.0001	48		ND	ND
Mesocricetus auratus	17.6	36.3	-2.6		0.1133	124.2		2.45	AnAge: The Animal

Microtus agrestis	1.9	20.7	-18.4		0.0001	31		2.3	Ageing and Longevity Database
Microtus arvalis	5.7	22.6	-13		0.0802	27.5	AnAge: The Animal Ageing and Longevity Database	1.85	
Microtus fortis	6.8	27.6	-16.4		0.0427	56.29	Luo et al (2000)	ND	ND
Microtus ochrogaster	8.4	28.1	-13.9		0.0001	37	Alhajeri et al. (2020)	3.02	AnAge: The Animal Ageing and Longevity Database
Neotoma lepida	11	30.2	-8.3		0.1604	125		8.45	
Ondatra zibethicus	2.1	22.9	-20.9		0.1384	829		22.72	
Peromyscus aztecus	20.2	27.9	11.9		0.0859	37		ND	ND
Peromyscus maniculatus bairdii	6.7	25.2	-14.1		0.3864	19		2	AnAge: The Animal Ageing and Longevity Database
Peromyscus nudipes	22.6	30.1	14.5		0.0687	39.83		ND	ND
Peromyscus polionotus	17.9	33.6	1.7		0.0001	14		1.68	AnAge: The Animal Ageing and
Phodopus	4.9	27.8	-20.8		0.0305	14		1.45	

roborovskii									Longevity Database
Sigmodon hispidus	16.7	33.8	-1.2		0.1102	99		6.2	
Jaculus jaculus	23.9	39.3	6.3		0.1080	55	AnAge: The Animal Ageing and Longevity Database	2	
Dipodomys ordii	11.8	30.4	-7.6		0.1085	44	Alhajeri et al. (2020)	5	
Acomys dimidiatus	25.2	39.1	9.4		999	45.2	AnAge: The Animal Ageing and Longevity Database	ND	ND
Acomys kempi	26.5	33.6	19.2		0.1872	49.3	Pinheiro et al(2018)	ND	ND
Acomys russatus	24.2	37.2	9.6		0.0614	37	Alhajeri et al. (2020)	7	AnAge: The Animal Ageing and Longevity Database
Hylomyscus alleni	25	30.8	18.4		0.0001	18		ND	ND
Lophiomys imhausi	22.4	31.7	12.5		0.2516	755		ND	ND

Meriones unguiculatus	1.8	25	-24.1		0.0439	53.2	AnAge: The Animal Ageing and Longevity Database	3	AnAge: The Animal Ageing and Longevity Database
Mus caroli	23.3	31.3	14		0.0001	11.2		1.2	
Mus minutoides	21.9	30.2	12.4		0.0798	7.2		0.97	
Mus musculus	12.9	28.6	-4.4		0.0001	16	Alhajeri et al. (2020)	1.25	
Mus pahari	19.4	28.8	8.3		0.0813	22		ND	ND
Mus spicilegus	9.2	24.6	-8.6		0.0001	19.9	AnAge: The Animal Ageing and Longevity Database	ND	ND
Mus spretus	15.3	29.2	1		0.0001	14	Alhajeri et al. (2020)	ND	ND
Psammomys obesus	22.1	37.8	4.5		0.0001	212	AnAge: The Animal Ageing and Longevity Database	6.25	AnAge: The Animal Ageing and Longevity Database
Rattus norvegicus	7.4	23.9	-10.5		0.0591	147	Alhajeri et al. (2020)	5.81	
Rhabdomys dilectus	18.5	29.2	6.2		0.0763	39		ND	ND
Rhynchomys soricoides	18.8	25.2	11.4		0.0811	170		ND	ND
Cricetomys	26.3	34.5	16.7		0.0953	1500	AnAge:	25.7	AnAge:

gambianus							The Animal Ageing and Longevity Database		The Animal Ageing and Longevity Database
Ictidomys tridecemlineatus	8.2	27.9	-14		0.0327	65	Alhajeri et al. (2020)	3.24	
Marmota marmota	5.8	18.2	-6.8		0.0001	4341		30	
Sciurus vulgaris	-0.8	20.9	-23.8		0.0695	600	AnAge: The Animal Ageing and Longevity Database	9.25	
Nannospalax galili	-	38	2.4		0.2403	140.5	Sumbera et al. (2023)	ND	ND
Cricetulus griseus	3.2	26.1	-22.4		0.0001	21.90	Alhajeri et al. (2020)	2	AnAge: The Animal Ageing and Longevity Database
Urocitellus parryii	-7.5	16	-29.9		0.6070	620		ND	ND
Dicrostonyx groenlandicus	-11.6	12.3	-32.4		0.2206	65		4.35	AnAge: The Animal Ageing and Longevity
Phodopus sungorus	2.1	25.1	-23		0.0958	23.4	AnAge: The Animal	2	

							Ageing and Longevity Database		Database
Marmota himalayana	0	18.4	-18.6		0.0001	5931.4	Alhajeri et al. (2020)	ND	ND

Note: ND means no data.

References

Alhajeri, B. H. et al, 2020, A global test of Allen's rule in rodents, Global Ecology and Biogeography, 29, 2248-60.

Sumbera, R. et al, 2023, Thermal biology in the Upper Galili Mountain blind mole rat (Nannospalax galili) and an overview of spalacine energetics, Journal of Thermal Biology, 115, 103618.

Thomas, H. G. et al, 2016, Burrow architecture of the Damaraland mole-rat (Fukomys damarensis) from South Africa, African Zoology, 51 (1) , 29-36

Luo Z.X. et al (2000). Zoology of China. Theroidea. Volume VI. Rodentia (Part II) Hamsters. Beijing: Science Press,1-522; http://www.zoology.csdb.cn/.

Pinheiro, G. et al, 2018, The African spiny mouse (Acomys spp.) as an emerging model for development and regeneration, Lab Animal, 52, 565-76.

Table S4 Correlation analyses between ω and body weight, ω and weight at birth.

	Body weight	Weight at birth
Raw data		
Pearson Correlation	-0.01	0.15
Sig.(2-tailed)	0.94	0.41
After controlling phylogenetic inertia		
Pearson Correlation	-0.08	-0.14
Sig.(2-tailed)	0.58	0.47

Supplementary text 1 All the intact *UCP1* gene sequences used in this study.

>*Homo sapiens*
ATGGGGGGCCTGACAGCCTCGGACGTACACCCGACCCTGGGGGTCCAGCTCTTCTCAGCTGGAATAGCGGCGTGCTTGGCGGACGTGATCACCTTCC
CGCTGGACACGGCCAAAGTCCGGCTCCAGGTCCAAGGTGAATGCCCGACGTCCAGTGTTATTAGGTATAAAGGTGTCCTGGGAACAATCACCGCTGT
GGTAAAAACAGAAGGGCGGATGAAACTCTACAGCGGGCTGCCTGCGGGGCTTCAGCGGCAAATCAGCTCCGCCTCTCTCAGGATCGGCCTCTACGAC
ACGGTCCAGGAGTTCCTCACCGCAGGGAAAGAAACAGCACCTAGTTTAGGAAGCAAGATTTTAGCTGGTCTAACGACTGGAGGAGTGGCAGTATTCA
TTGGGCAACCCACAGAGGTCGTGAAAGTCAGACTTCAAGCACAGAGCCATCTCCACGGAATCAAACCTCGCTACACGGGGACTTATAATGCGTACAG
AATAATAGCAACAACCGAAGGCTTGACGGGTCTTTGGAAAGGGACTACTCCCAATCTGATGAGAAGTGTCATCATCAATTGTACAGAGCTAGTAACAT
ATGATCTAATGAAGGAGGCCTTTGTGAAAAACAACATATTAGCAGATGACGTCCCCTGCCACTTGGTGTCGGCTCTTATCGCTGGATTTTGCGCAACAG
CTATGTCCTCCCCGGTGGATGTAGTAAAAACCAGATTTATTAATTCTCCACCAGGACAGTACAAAAGTGTGCCCAACTGTGCAATGAAAGTGTTCACTA
ACGAAGGACCAACGGCTTTCTTCAAGGGGTTGGTACCTTCCTTCTTGCGACTTGGATCCTGGAACGTCATTATGTTTGTGTGCTTTGAACAACTGAAA
CGAGAACTGTCAAAGTCAAGGCAGACTATGGACTGTGCCACATAA

>*Microtus ochrogaster*
ATGGTGAGCCCAACAACTTCCGAAGTGCACCCCACCATGGGGGTCAAGATCTTCTCAGCTGGCATATCCGCCTGCCTGGCAGATATCATCACCTTCCCA
CTGGACACAGCCAAAGTCCGGCTTCAGATCCAAGGTGAAGGCCAGACCTCCACTACCATTAGGTATAAAGGTGTCCTGGGAACCATCACCACCCTGG
CAAAAACAGAAGGGTTGCCGAAACTGTACAGCGGTCTGCCTGCTGGCATTCAGAGGCAAATCAGCTTCGCTTCACTCAGGATCGGTCTCTATGATACT
GTTCAAGAGTACTTCTCTTCAGGCAAAGAAACGCCTCCCACTTTGGGAAACAGGATCTCAGCTGGCTTATTGACTGGAGGTGTGGCAGTATTCATCGG
GCAACCTACCGAGGTCGTGAAAGTCAGACTCCAAGCACAGAGTCACTTACACGGGATCAAACCCCGCTACACAGGGACCTACAATGCTTACAGAATT
ATAGCCACAACAGAAAGCTTATCAACCCTCTGGAAAGGGACAACCCCTAATCTGATGAGAAATGTCATTATCAATTGTACAGAGCTGGTAACATATGAC
CTCATGAAGGGGGCCCTTGTGAACAACCAAATACTGGCAGACGACGTGCCCTGCCATTTACTGTCAGCTCTTGTGGCCGGGTTTTGCACCACCTTCCT
GGCCTCTCCGGCAGATGTGGTAAAAACAAGATTCATCAACTCTCTACCAGGACAGTACCCGAGTGTACCCAGCTGTGCTATGACCATGCTCACCAAGG
AAGGACCAACAGCTTTTTTCAAAGGGTTTGTGCCTTCTTTCCTGCGACTGGCGTCCTGGAATGTCATCATGTTTGTGTGCTTCGAACAGCTGAAGAAG
GAATTGATGAAGTCCAGGCAGACGGTGGACTGCACCACATAA

>*Mus spicilegus*
ATGGTGAGCCCGACAACTTCCGAAGTGCAACCCACCATGGGGGTCAAGATCTTCTCAGCTGGAGTTTCAGCTTGCCTGGCAGATATCATCACCTTCCC
GCTGGACACTGCCAAAGTCCGCCTTCAGATCCAAGGTGAAGGCCAGGCTTCCAGTACCATTAGGTATAAAGGTGTCCTAGGGACCATCACCACCCTGG
CAAAAACAGAAGGATTACCGAAACTGTACAGCGGTCTGCCTGCAGGCATTCAGAGGCAAATCAGCTTTGCCTCACTCAGGATTGGCCTCTACGACTC
GGTCCAAGAGTACTTCTCTTCAGGGAGAGAAACACCTGCCTCTCTCGGAAACAAGATCTCAGCCGGCTTAATGACTGGAGGTGTGGCAGTGTTCATT
GGGCAGCCTACAGAGGTCGTGAAGGTCAGAATGCAAGCCCAGAGCCATCTGCATGGGATCAAACCCCGCTACACAGGGACCTACAATGCTTACAGAG
TTATAGCCACCACAGAAAGCTTGTCAACACTTTGGAAAGGGACGACCCCTAATCTAATGAGAAATGTCATCATCAATTGTACAGAGCTGGTAACATATG
ACCTCATGAAGGGGGCCCTTGTAAACAACAAAATACTGGCAGATGACGTCCCCTGCCATTTACTGTCAGCTCTTGTTGCCGGGTTTTGCACCACACTC
CTGGCCTCTCCAGTGGATGTGGTAAAAACAAGATTCATCAACTCTCTGCCAGGACAGTACCCAAGCGTACCAAGCTGTGCGATGTCCATGTACACCAA
GGAAGGACCGACGGCCTTTTTCAAAGGGTTTGTGGCTTCTTTTCTGCGACTCGGGTCCTGGAACGTCATCATGTTTGTGTGCTTTGAACAGCTGAAAA
AAGAGCTGATGAAGTCCAGGCAGACAGTGGATTGTACCACATAA

>*Rattus norvegicus*
ATGGTGAGTTCGACAACTTCCGAAGTGCAACCCACCATGGGGGTCAAGATCTTCTCAGCCGGCGTTTCTGCCTGCCTAGCAGACATCATCACCTTCCC
GCTGGACACCGCCAAAGTCCGCCTTCAGATCCAAGGTGAAGGCCAGGCTTCCAGTACTATTAGGTATAAAGGTGTCTTAGGGACCATCACCACCCTGG
CCAAGACAGAAGGATTGCCGAAACTGTACAGCGGTCTGCCTGCTGGCATCCAGAGGCAAATCAGCTTTGCTTCCCTCAGGATTGGCCTCTACGATACG
GTCCAAGAGTACTTCTCTTCAGGGAGAGAAACGCCTGCCTCTTTGGGAAGCAAGATCTCGGCTGGCTTGATGACGGGTGGCGTGGCGGTATTCATTGG
GCAGCCCACAGAGGTGGTGAAGGTCAGAATGCAAGCACAAAGCCATCTGCACGGGATCAAACCCCGCTACACTGGGACCTACAATGCTTACAGAGTT
ATAGCCACCACAGAAAGCTTGTCAACACTGTGGAAAGGGACGACTCCTAATCTAATGAGAAATGTCATCATCAACTGTACAGAGCTGGTGACATATGA
CCTCATGAAGGGGGCCCTTGTGAACCACCACATACTGGCAGATGACGTCCCCTGCCATTTACTGTCAGCTCTTGTCGCCGGGTTTTGCACCACACTCCT
GGCCTCTCCGGTGGATGTGGTAAAAACGAGATTCATCAACTCTCTACCAGGACAGTACCCAAGTGTACCCAGCTGTGCAATGACCATGTACACCAAGG
AAGGACCGGCAGCCTTTTTCAAAGGGTTTGCGCCTTCTTTTCTGCGACTCGGATCCTGGAACGTCATCATGTTTGTGTGCTTTGAACAGCTGAAGAAA
GAGCTGATGAAGTCCCGGCAGACAGTGGACTGCACCACATAG

>*Urocitellus parryii*
ATGGTGAGCCCCACAGCCTCCGACGTGCACCCGACCATGGCCATCAAGATCGTCTCAGCCGGAGTGTCAGCCTGCTTGGCGGATGTGATCACCTTCCC

GCTGGACACCGCCAAAGTCCGGCTACAGATCCAAGGCGAATTCCCAGTCTCCAGCGGTATTACGTATAAAGGTGTCCTGGGAACAATCACCACCCTAG
CAAAAACCGAAGGGCCCATGAAACTGTACAGCGGGTTGCCTGCGGGATTGCAAAGGCAAATAAGCTTCGCCTCTCTTAGGATCGGCCTCTATGATTCT
GTCCAGGAGTACTTCACCTCAGGGAATGAAACAGCACCCAGTTTGGGAAGCAAAATCTCAGCCGGACTCACAACTGGAGGAGTGGCAGTGTTCATTG
GGCAGCCCACCGAGGTGGTGAAGGTCAGGCTCCAAGCACAGAGCCACCTACATGGTCTCAAACCTCGCTACACCGGGACCTACAACGCGTACAGAA
TTATAGTAACAACAGAGAGCTTCAGGAGTCTTTGGAAAGGGACTACTCCTAATCTGTTGAGAAACGTCATCATCAATTGCACAGAGTTGGTAACATATG
ACCTGATGAAGGGGGCTCTTGTGAGAAACAAAATCCTAGCAGATGATGTCCCCTGCCACCTGCTGTCCGCTTTCGTCGCTGGATTTTGCACCACACTC
CTGTCCTCGCCTGCTGATGTAGTGAAGACCAGATTTATTAACTCTCCGCCAGGGCAGTATACCAGCGTGCCCAGATGTGCAATGGCAATGCTCCACCAG
GAAGGGCCGTCGGCATTTTTCAAAGGATTTCTACCTTCCTTCCTGCGACTGGCATCCTGGAACCTCATTATGTTCGTGAGCTTTGAACAGCTGAAACGA
GAATTGATGAAGTCCAGGCAGACAGTGGACTGCGCCACATGA

>*Nannospalax galili*
ATGGTGAGCCCGACAACCTCGGAAGCGGCCCCCACCTTGGGGGTCAAGATCTTCTCAGCCGGCGTGGCTGCCTGCCTAGCCGATATCATCACCTTCCC
GCTGGACACCGCCAAAGTCCGGCTCCAGATCCAAGGTGAAAGCCAGATCTCCAGTGGCATTAGGTATAAAGGTGTTCTGGGAACAATTACCACTCTG
GCAAGAACCGAAGGGCTGATGAAACTGTACAGCGGGCTGCCAGCAGGAATTCAGAGGCAGATCAGCTTTGCCTCGCTCAGGATTGGCCTCTATGATT
CTGTCCAAGAGTACTTCTCTTCGGGGAAAGAAACACCACCAACTTTGGGAAACAAGATCTCAGCTGGCTTAATGACTGGAGGGGTGGCAGTATTCAT
CGGGCAACCCACAGAGGTCGTGAAAGTCAGACTCCAAGCACAGAGCCACCTACATGGGATCAAGCCCCGCTACACTGGGACCTACAATGCTTACAGA
ATTATAGCAACAACAGAAAGCTTCTTCACCCTCTGGAAAGGGACTACCCCTAATCTAATGAGAAATATCATCATCAATTGTACAGAGCTAGTAACATATG
ACCTCATAAAGGGAGCCCTTGTGAACAACCAAATACTAGCAGATGATGTCCCATGCCATTTACTGTCAGCTCTCATTGCTGGGTTTTGCACAACACTCC
TGGCCTCTCCAGTGGATGTGGTAAAAACAAGATTCATCAATTCTGTACCAGGACAGTACACAAGTGTGCCCAGCTGTGCAATGACAATGTTCACTAAG
GAAGGACCTAAGGCTTTTTTCAAAGGGTTTGTGCCTTCTTTCCTGCGACTTGGGTCCTGGAACGTCATCATGTTTGTGTGCTTTGAACAGCTGAAAAA
AGAATTGATGAAGTCAAGGCAGACTGTGGACTGCACCACATAA

>*Ictidomys tridecemlineatus*
ATGGTGAGCCCCACGGCCTCCGACGTGCACCCGACCATGGGCATCAAGATCGTCTCAGCCGGAGTGTCAGCCTGCTTGGCGGATGTGATCACCTTCCC
GCTGGACACCGCCAAAGTCCGGCTACAGATCCAAGGCGAATTCCCTGTCTCCAGCGGTATTACGTATAAAGGAGTCCTGGGAACAATCACCACCCTGG
CCAAAACCGAAGGGCCCATGAAACTGTACAGCGGGCTGCCCGCGGGACTGCAAAGGCAAATCAGCTTCGCCTCTCTTAGGATCGGCCTCTACGATTC

TGTCCAGGAGTTCTTCACCTCAGGGGATGAAACAGCACCCAGCTTGGGAAGCAAAATCTCAGCCGGACTCACAACTGGAGGAGTGGCAGTGTTCATT
GGGCAGCCCACCGAGGTGGTGAAGGTCAGGCTCCAAGCACAGAGCCACCTACACGGTCTCAAACCTCGCTACACCGGGACCTACAACGCGTACAGA
ATTATAGCAACAACAGAGAGCTTCAGGAGTCTTTGGAAAGggaCTACTCCTAATCTGTTGAGAAATGTCATCATCAATTGCACAGAGTTGGTAACATATG
ACCTGATGAAGGGGGCTCTTGTGAGAAACAAAATCCTAGCAGATGATGTCCCCTGCCACCTGCTGTCCGCTTTCGTCGCTGGATTTTGCACCACACTC
CTGTCCTCGCCTGCTGATGTAGTGAAGACCAGATTTATTAACTCTCCGCCAGGGCAGTATACCAGCGTGCCCAGATGTGCAATGACAATGCTCCACCAG
GAAGGGCCGTCGGCATTTTTCAAAGGATTTCTACCTTCCTTCCTGCGACTGGCATCCTGGAACCTCATTATGTTCGTGAGCTTTGAACAGCTGAAACGA
GAATTGATGAAGTCCAGGCAGACAGTGGACTGCGCCACATGA

>*Mus pahari*
ATGGTGAGCCCAACAACTTCTGAAGTGCAACCCACCATGGGGGTCAAGATCTTCTCAGCCGGAGTTTCTGCCTGCCTGGCAGATATCATCACCTTCCC
GCTGGACACCGCCAAAGTCCGCCTTCAGATCCAAGGTGAAGGCCAGGCTTCCAGTACCATTAGGTATAAAGGTGTCCTCGGGACCATCACCaccctggcaa
aaacagaaggaTTGCCGAAACTGTACAGCGGTCTGCCTGCGGGCATTCAGAGGCAAATCAGCTTTGCCTCACTCAGGATTGGCCTCTACGACTCGGTCCAA
GAGTACTTCTCTTCAGGGACAGAAACACCCGCCTCTCTGGGAAACAAGATCTCAGCCGGCTTAATGACTGGAGGCGTGGCAGTGTTCATTGGGCAGC
CTACAGAGGTCGTGAAGGTCAGGATGCAAGCCCAGAGCCATCTGCACGGGATCAAACCCCGCTACACCGGGACCTACAATGCTTACAGAGTTATAGC
CACCACAGAAAGCTTGTCAACACTTTGGAAAGGGACGACCCCTAATCTCATGAGAAATGTCATCATCAATTGCACAGAGCTGGTAACATATGACCTCA
TGAAGGGGGCCCTTGTAAACAACAAAATACTGGCAGATGACGTCCCCTGCCATTTACTGTCAGCTCTTGTTGCCGGGTTTTGCACCACACTCCTGGCC
TCTCCCGTGGATGTGGTAAAAACAAGATTCATCAACTCTCTGCCAGGACAGTACCCAAGCGTACCGAACTGTGCAATGACCATGTACAGGAAGGAAG
GACCGACGGCCTTCTTCAAAGGGTTTGTGGCTTCTTTTCTGCGACTCGGGTCCTGGAATGTCATCATGTTTGTGTGCTTTGAACAGCTGAAAAAGGAA
CTGATGAAGTCCAGGCAGACAGTGGATTGCTCCACATGA

>*Mus caroli*
ATGGTGAGCCCGACAACTTCCGAAGTGCAACCCACCATGGGGGTCAAGATCTTCTCAGCCGGAGTTTCAGCTTGCCTGGCAGATATCATCACCTTCCC
GCTGGACACTGCCAAAGTCCGCCTTCAGATCCAAGGTGAAGGCCAGGCTTCCAGTACCATTAGGTATAAAGGTGTCCTAGGGACCATCACCaccctggcaaa
aacagaaggaTTGCCGAAGCTGTACAGCGGTCTGCCTGCGGGCATTCAGAGGCAAATCAGCTTTGCCTCACTCAGGATTGGCCTCTACGACTCGGTCCAAG
AGTACTTCTCTTCGGGGAGAGAAACACCTGCCTCTCTTGGAAACAAGATCTCAGCCGGCTTAATGACTGGAGGTGTGGCAGTGTTCATTGGGCAGCCT
ACAGAGGTCGTGAAGGTCAGAATGCAAGCCCAGAGCCATCTGCATGGAATCAAACCCCGCTACACGGGGACCTACAATGCTTACAGAGTTATAGCCA

CCACAGAAAGCTTGTCGACACTTTGGAAAGGGACGACCCCTAATCTAATGAGAAATGTCATCATCAATTGTACAGAGCTGGTAACATATGACCTCATG
AAGGGGGCCCTTGTAAACAACAAAATACTGGCAGATGACGTCCCCTGCCATTTACTGTCAGCTCTTGTTGCCGGGTTTTGCACCACACTCCTGGCCTC
TCCAGTGGATGTGGTAAAAACAAGATTCATCAACTCTCTGCCAGGACAGTACCCAAGCGTACCGAGCTGTGCGATGTCCATGTACACCAAGGAAGGA
CCGACGGCCTTTTTCAAAGGGTTTGTGGCTTCTTTTCTGCGACTCGGGTCCTGGAACGTCATCATGTTTGTGTGCTTTGAACAGCTGAAAAAAGAGCT
GATGAAGTCCAGGCAGACAGTGGATTGCACCACATAA

>*Peromyscus maniculatus bairdii*
ATGGTGAGCCCGACAACTTCCGAAGTGCCTCCTACCATGGGGGTCAAGATCTTCTCAGCCGGCTTGGCCGCCTGTCTGGCAGATATCATCACCTTCCCG
CTGGATACAGCCAAAGTCCGGCTTCAGATCCAAGGTGAAGGCCAGAACTCTAGTACCATTAGGTATAAAGGTGTCCTGGGGACCATCACCACCCTGGC
AAAAACAGAAGGGTTGCCGAAACTGTACAGCGGTCTGCCTGCTGGCATTCAGAGGCAAATCAGCTTTGCCTCACTCAGGATCGGCCTCTACGATACT
GTCCAAGAGTACTTCTCTTCAGGGAGAGAAACGCCCCCCACTTTGATAAACAGGATCTCAGCTGGCTTGATGACTGGAGGTGTGGCAGTATTCATCGG
GCAACCTACAGAGGTCGTGAAAGTCAGACTCCAAGCACAGAGCCACCTGCATGGGATCAAACCCCGCTACACTGGGACCTACAATGCTTACAGAATT
ATAGCTACAACAGAAAGCTTCTCAACACTCTGGAAAGGGACAAGCCCTAATCTGATCAGGAATATCATCATCAATTGTACAGAGCTGGTAACATATGAC
CTCATGAAGGGGGCCCTTGTGAACAACCAAATTTTGGCAGATGACGTTCCCTGCCACTTACTGTCAGCTCTTGTCGCCGGGTTTTGCACCACACTCCT
GGCCTCTCCGGCGGATGTGGTAAAAACAAGATTCATCAACTCTCTGCCGGGACAGTACCCAAGTGTGCCCAGCTGTGCAATGACCATGTTCACCAACG
AAGGACCAACAGCTTTCTTCAAAGGGTTTGTGCCCTCTTTCCTGCGACTCGCATCCTGGAATGTCATCATGTTCGTGTGCTTTGAACAGCTGAAGAAA
GAACTGATGAAGTCAAGGCAGACAGTGGACTGCTCCACATAA

>*Heterocephalus glaber*
ATGGTGAGCCCGACGGCGTCGGAGGTGCACCCCACCATGGGGGTCAAGATCTTCTCCGCCGGAGTGGCGGCCTGTTTGGCCGATGTGATCACCTTTCC
GCTGGACACGGCCAAAGTCCGCCTGCAGATTCAAGGCGAAAGCCAGATCTCCAGCGGTATTCAGTATAAAGGTGCCCTGGGAACTATCACCACTCTG
GCAAAGACCGAAGGGCCCATGAAACTATACAGCGGGCTGCCCGCGGGCCTGCAGAGGCAAATAAGCTTCGCCTCTCTCAGGATCGGCCTCTATGACA
CGGTCCAGGAGTTCTACACCTCGGAGAAAGATATAACACCTAGTTTGGGAAGTAGGATCGCAGCTGGCTTAACCACTGGAGGAGTGGCTGTATTCATC
GGGCAGCCCACAGAGGTCGTGAAAGTCAGACTCCAAGCACAGAGCCAACTACATGGCCTCAAACCACGCTACACTGGGACTTACAATGCTTACCGAA
TTATAGCAACAACAGAAAGCTTGAAAAGTCTTTGGAAAGgGACTACTCCCAATCTATTGAGAAATATTATCATCAATTGCACGGAGCTAGTAACATATG
ACCTGATGAAGGGTGCCCTTGTGAGAAACAAGATACTAGCAGATGATGTCCCCTGCCACTTACTGTCTGCTCTTATCGCTGGGTTTTGCACAACGCTTC

TGTCCTCTCCAGTGGATGTGGTGAAAACTAGATTTATTAATTCTCCACCAGGACAGTACATAAGTGTGCCCAGCTGTGCAATGACCATGCTCAAAAAGG
AACGATGGACAGCTTTTTTCAAAGGATTTGTGCCTTCGTTCCTGCGACTTGCATCCTGGAATGTTATTATGTTTGTGTGCTTTGAGCAACTGAAGCGTG
AGTTGACGAAGTCAAGGCAGCCCACAGACTACGCCACATAA

>*Mus musculus*
ATGGTGAGCCCGACAACTTCCGAAGTGCAACCCACCATGGGGGTCAAGATCTTCTCAGCCGGAGTTTCAGCTTGCCTGGCAGATATCATCACCTTCCC
GCTGGACACTGCCAAAGTCCGCCTTCAGatcCAAGGTGAAGGCCAGGCTTCCAGTACCATTAGGTATAAAGGTGTCCTAGGGACCATCACCaccctggcaaaa
acagaaggaTTGCCGAAACTGTACAGCGGTCTGCCTGCGGGCATTCAGAGGCAAATCAGCtttgcctcactcaggattgGCCTCTACGACTCAGTCCAAGAGTACTTC
TCTTCAGGGAGAGAAACACCTGCCTCTCTCGGAAACAAGATCTCAGCCGGCTTAATGACTGGAGGTGTGGCAGTGTTCATTGGGCAGCCTACAGAGG
TCGTGAAGGTCAGAATGCAAGCCCAGAGCCATCTGCATGGGATCAAACCCCGCTACACGGGGACCTACAATGCTTACAGAGTTATAGCCACCACAGA
AAGCTTGTCAACACTTTGGAAAGGGACGACCCCTAATCTAATGAGAAATGTCATCATCAATTGTACAGAGCTGGTAACATATGACCTCATGAAGGGGG
CCCTTGTAAACAACAAAATACTGGCAGATGACGTCCCCTGCCATTTACTGTCAGCTCTTGTTGCCGGGTTTTGCACCACACTCCTGGCCTCTCCAGTGG
ATGTGGTAAAAACAAGATTCATCAACTCTCTGCCAGGACAGTATCCAAGCGTACCAAGCTGTGCGATGTCCATGTACACCAAGGAAGGACCGACGGC
CTTTTTCAAAGGGTTTGTGGCTTCTTTTCTGCGACTCGGGTCCTGGAACGTCATCATGTTTGTGTGCTTTGAACAGCTGAAAAAAGAGCTGATGAAGT
CCAGGCAGACAGTGGATTGTACCACATAA

>*Meriones unguiculatus*
ATGGTGAGCCCGACAACTTCTGAAGTGCAGCCCACCATGGGGGTCAAGATCTTCTCAGCCGGTGTGGCGGCCTGCCTGGCAGACATCATTACCTTCCC
GCTGGACACCGCCAAAGTCCGCCTTCAGATCCAAGGTGAAGGACCAGCCTCCAGCACCATTCGGTATAAAGGTGTCCTAGGGACCATCAGCACCCTG
GCAAAAACAGAAGGGGTGCCGAAACTGTATAGCGGTCTGCCTGCGGGCATTCAGAGGCAAATCAGCTTCGCCTCACTCAGGATTGGCCTCTACGATA
CTGTCCAAGAGTACTTCGCTTCAGGGAGAGAAACACCTGCCACTTTGGGGAACAAGATCTCGGCTGGCTTAATGACTGGAGGTGTGGCAGTGTTCAT
CGGGCAACCTACAGAAGTCGTGAAAGTCAGAATGCAAGCACAGAGCCATCTACACGGCATCAAACCCCGCTACACTGGGACCTACAATGCTTACAGA
ATTATAGCCACAACAGAAAGCTTGTCAACACTCTGGAAAGGGACAACCCCTAATCTAATGAGAAATGTCATCATCAATTGTACAGAGCTGGTAACATAT
GACCTCATGAAAGGGGCCCTGGTGAATAACCGAATACTGGCAGATGACGTTCCATGCCATTTGCTGTCAGCACTTGTTGCCGGCTTTTGCACCACACT
CCTGGCCTCTCCAGTGGATGTGGTGAAAACGAGATTCATCAACTCTCTACCAGGACAGTACCCGAGTGTGCCCAGCTGTGCAATGACCATGTTCAACA
AGGAAGGACCAACGGCCTTCTTCAAAGGGTTTGTGCCTTCTTTCCTGCGACTGGGGTCCTGGAACGTCATCATGTTTGTGTGCTTTGAGCAGCTGAAA

AAAGAACTGATGAAGTCAAGGCAGACAGTGGACTGCACCACATAA

>*Dipodomys ordii*
ATGGCGCCGGCCGCCGCCCCGGAGGAGCACCCGACCATGGGCGTCCAGATGTTCTCGGCGGGGGTGTCCGCCTGCCTGGCCGACATCATCACCTTCC
CACTGGACACCGCCAAAGTGCGCCTGCAGATTCAAGGGGAAAGCCCTACGTCCAGCGGCATCAAGTACAAGGGGGTCCTGGGAACAATCACCACGC
TGGCCAAAACCGAAGGCCCCGCGAAACTGTACAGCGGGCTGCCCGCCGGCATCCAGAGGCAGATCAGCTCGGCCTCGCTGCGCATCGGCCTCTATGA
AACCGCCCAGGAGTACTTCGCGGCCGAGACCGACAAACCACCAAGTTTGGGAAACAAGATCTTAGCTGGCTTGATGACCGGAAGCGTGGCCGTGTTT
GTCGGGCAACCCACGGAGGTGGTGAAGGTCCGAATGCAAGCACAGAGCCACCTCCACGGCCTCAAGCCTCGCTACACGGGCACCTACAACGCGTAC
AGAATTATCGCCACCAAAGAGAGCCTGTCGACGCTTTGGAAAGGGACCTCACCTAACCTGCTGAGAAGCGTCATCATCAACTGCACAGAGCTTGTGA
CCTACGACCTGGTGAAGGATTTCTTCGTGGGAAACAAAATCCTAGCAGACGATGTCCCCTGCCACTTACTGTCGGCTCTTATTGCTGGATTCTCCGCAA
CGCTCATGTCGTCTCCAGCGGATGTGGTGAAAACCAGATTCATTAATTCCCCGCCAGGACAGTACCTGAGCGTGCCCAGCTGTGCCATGAGCATGCTT
ACCAAGGAAGGGCCGACAGCTTTCTTCAAAGGATTCATGCCTTCCTTCCTGCGACTGGCATCCTGGAACGTCATTATGTTTGTGTGCTTCGAGCAGCTC
AAACGAGAGCTGACCAAGTCCAGGCAGACTGTGGACTGCACCACCTAG

>*Fukomys damarensis*
ATGGGGAGCCCGACGGCGTCGGAGGTGCACCCCACCGTGGGGGTCAAGATCTTCTCCGCCGGAGTGTCAGCCTGCTTGGCGGACGTGATCACTTTCC
CGCTGGACACCGCCAAAGTCCGCCTGCAGATCCAAGGTGAAAGCCAGATCTCCAGCGGTATTCGGTATAAAGGGGTCCTGGGAACTATCACCACTCT
GGCGAAGACCGAAGGGCCCGTGAAGCTGTACAGCGGGCTGCCCGCCGGCCTGCAGAGGCAAATAAGCTTCGCCTCCCTCAGGATCGGCCTCTATGAC
ACCGTCCAGGAGTTCTACACCTCGGAGAAAGACACAACACCTAGTTTGGGAAATAGGATCGCAGCTGGCTTAACCACTGGAGGAGTGGCAGTATTCA
TCGGACAGCCCACAGAGGTCGTGAAAGTCAGACTCCAGGCACAGAGCCATCTTCACGGCCTCAAACCACGCTACACTGGGACTTACAATGCTTACAG
AATTATAGCTACAACAGAAAGCTTGAAAAGTCTTTGGAAAGggACTACTCCCAATCTGTTGAGAAATATTATCATCAATTGCACGGAGCTAGTAACGTAT
GACCTGATGAAGGGCGCCCTTGTGAGAAACAAGATACTAGCAGATGACATCCCCTGCCACTTACTGTCTGCTCTTATTGCTGGGTTTTGCACAACGCTT
CTGTCCTCTCCTGTGGATGTGGTGAAAACCAGATTTATTAATTCTGCACCGGGACAGTATGTAAGTGTGCCCAGCTGCGCGATGACCATGCTCAACAAG
GAAGGACCAACAGCTTTTTTCAAGGGATTTGTGCCTTCCTTCCTGCGCCTCGCATCCTGGAACGTTATTATGTTCGTTTGCTTCGAGCAGCTGAAGCGA
GAGCTGACGAAGTCAGGGCAGTCCGCGGACTACGCCGCCTAG

>*Jaculus jaculus*
ATGGTGAGCCAGACAGCCACGGACGTGCCCCCTCCCACCATGGGGGTCAAGATCGTCTCCGCCGGGGTGGCCGCCTGCCTGGCAGACGTGATCACCT
TCCCGCTGGACACTGCCAAAGTCCGACTGCAGATCCAAGGCGAGGCTCCGAACTCCAGCGGCATCAAGTACAAAGGCGTCCTGGGAACCGTCACCA
CGCTGGCGAGGACCGAAGGGCCGGCGAAGCTGTACAGCGGGCTGCCCGCCGGCATTCAGAGGCAAATAAGCTCCGCCTCCCTCAGGATCGGCCTGTA
CGATTCGGTCCAGGAGTACTTCTCGGAAGGGAAAGAAACAACACCCAGTTTGGGAAATAAGATGTGCGCTGGCTTAATGACCGGAGGGGTGGCAGTG
TTCATTGGACAGCCCACAGAGGTGGTAAAAGTGAGGCTCCAAGCGCAGAGCCACCTGCATGGCATCAAACCCCGTTACACCGGAACTTACAACGCCT
ACAGAATTATTGCAACGACAGAAAGCTTTTCGACCCTATGGCGTGGGACTACCCCTAACCTGTTGAGAAATGTCATCATCAATTGCACAGAGCTGGTG
ACGTATGACCTGATGAAGGGAGCCCTTGTGAACAACCAAATATTAGCAGATGATGTCCCATGCCACTTGCTGTCTGCTTTGGTTGCTGGATTTTGCACA
ACATTCCTGGCTTCTCCAGCGGATGTGGTGAAGACAAGATACATCAATTCTCCAGCGGGTCAGTACCACAGCGTGCCCAGCTGCGCAATGGCAATGCT
CCAAAAAGAAGGGCCAACGGCGTTTTTCAAAGGATTCGTGCCTTCCTTCCTGCGTCTGGCCTCCTGGAATGTCATTATGTTCGTGTGCTTTGAGCAGCT
GAAGCGAGAACTGATGAAGTCAAGGCAGACGGTGGACTGTACCACATAA

>*Mesocricetus auratus*
ATGGTGAACCCAACAACTTCTGAAGTGCACCCCACCATGGGGGTCAAGATCTTCTCAGCGGGTGTGGCGGCCTGCCTGGCAGACATCATCACCTTCCC
GCTGGACACAGCCAAAGTCCGGCTTCAGATACAAGGTGAAGGCCAGATCTCCAGTACCATTAGGTATAAAGGTGTCCTGGGGACCATCACCACCCTG
GCAAAAACAGAAGGGTTGCCGAAACTGTACAGTGGTCTTCCTGCTGGTATCCAGAGACAAATCAGCtttgcctcactcaggattggCCTCTACGATACTGTCCAGG
AGTACTTCTCTTCAGGGAAAGAGACGCCTCCCACTTTGGGAAACAGGATCTCAGCTGGCTTAATGACCGGAGGTGTGGCGGTATTCATCGGGCAACCT
ACCGAGGTCGTGAAAGTCAGACTCCAAGCACAGAGCCACCTACACGGGATCAAACCCCGCTACACTGGGACCTACAATGCTTATAGAATTATAGCCAC
AACAGAAAGCTTTTCAACACTCTGGAAAGGGACGACCCCTAACCTGTTGAGGAATGTCATCATCAACTGTGTAGAGCTGGTAACATATGACCTCATGA
AGGGGGCCCTTGTGAACAACCAAATACTGGCAGATGACGTGCCATGCCATTTACTGTCAGCTTTTGTCGCTGGGTTTTGCACCACATTCCTGGCCTCTC
CGGCGGACGTAGTGAAAACGAGATTCATCAACTCTCTGCCAGGACAGTACCCAAGTGTACCCAGCTGTGCAATGACCATGCTCACCAAGGAAGGACC
AACAGCTTTTTTCAAAGGGTTCGTGCCTTCTTTCCTGCGGCTGGCGTCCTGGAATGTCATCATGTTTGTGTGCTTTGAACAGCTGAAGAAGGAGCTGT
CTAAGTCGAGGCAGACGGTGGACTGCACCACATAA

>*Cavia porcellus*
ATGGTGAGCCCGACGGTGTCAGACGTGCACCCCACCATGGGGGTCAAGATCTTCTCAGCCGGAGTGTCGGCCTGCTTGGCGGATGTGATCACCTTCCC

GCTGGACACCGCCAAAGTCCGCCTACAGATCCAAGGCGAAAGCCCGACCTCCAGCGGCATTCGGTATAAGGGCGTCCTGGGGACTATCACCACGCTG
GCAAAGACTGAAGGGCCCGTGAAACTGTACAGCGGGCTCCCCGCAGGCCTGCAGAGGCAGATCAGCTTCGCCTCCCTCAGGATCGGCCTGTATGACA
CCGTCCAGGAGTTCTACACCTCGGAGAAAGATGCAACGCCTAGTTTGGGAAGCAGGATTGCAGCTGGCTTAACCACCGGAGGAGTGGCAGTATTCAT
CGGGCAGCCCACGGAGGTCGTGAAGGTCCGACTCCAGGCGCAGAGCCATCTACATGGCCTCAAACCTCGCTACACTGGGACTTACAATGCTTACAGA
ATTATAGCAACAACAGAAAGTTTGAAAAGTCTTTGGAAAGGGACAACTCCCAATCTGTTGAGAAATATCATCATCAACTGCACAGAGTTAGTCACGTA
CGACCTGATGAAGGGCGCCCTTGTGAGAAACAAGATACTAGCAGATGACGTCCCCTGCCATTTACTGTCAGCTCTTATTGCTGGGTTTTGCACAACGC
TTCTGTCCTCTCCAGTGGATGTGGTGAAAACCAGGTTTATTAATTCTCCACCGGGACAGTACCTAAGTGTGCCCAGCTGTGCAATGACAATGCTCCTCA
AGGAAGGACCCACGGCTTTTTTCAAAGGATTCGTACCTTCCTTCCTGCGACTGGCATCCTGGAACGTCATTATGTTTGTGTGCTTTGAGCAGCTGAAGA
GGGAGTTGATGAAGTCAAGGCAGCCCGTGGACTACGCCACTTAA

>*Chinchilla lanigera*
ATGGTGAGCCCGGCGACGTCGGACGTGCACCCCACCATGGGTGTCAAGATCTTCTCAGCCGGGGTGTCGGCCTGCTTGGCGGATGTGATCACCTTCCC
GCTGGACACGGCCAAAGTCCGCCTACAGATCCAGGGTGAAGGCCCGACCTCCAGCGCTATTCGGTATAAAGGCGTCCTAGGAACAATCACCACTCTG
GCAAAGACCGAAGGGCCCGTGAAACTGTACAGCGGGCTCCCCGCAGGCCTGCAGAGGCAGATAAGCTTCGCCTCGCTCAGAATCGGCCTCTATGACA
CCGTGCAGGAGTTCTACACCTCCGAGAACGATACAACACCTAGTTTGGGAAGTAGGATCGCAGCTGGCTTAACCACTGGAGGAGTGGCAGTATTCATC
GGGCAGCCCACAGAGGTCGTGAAAGTCCGACTCCAGGCACAGAGCCATCTACATGGCCTCAAACCTCGCTACACGGGGACTTACAATGCTTACAGAA
TTATAGCAACAACAGAAAGCTTGAAAAGTCTTTGGAAAGggaCTACTCCCAATCTATTGAGAAATATCATCATCAACTGCACAGAGCTAGTAACGTATGA
CCTGATGAAGGGCGCTCTTGTGAGAAACAAGATACTAGCAGATGACGTCCCCTGCCATTTACTGTCGGCTCTTATCGCTGGGTTTTGCACAACGCTTCT
GTCCTCTCCGGTGGATGTGGTGAAAACCAGATTTATTAATTCTCCACCAGGACAGTACATAAGTGTGCCCAGCTGTGCACTGACAATGCTCAACAAGG
AAGGACCAACGGCCTTTTTCAAAGGATTTGTACCTTCCTTCCTGCGACTCGCCTCCTGGAACGTTATTATGTTTGTGTGCTTTGAGCAGCTGAAGCGTG
AGTTGATGAAGTCAAGGCAGCCCGTGGACTATGCCACATAA

>*Acomys dimidiatus*
ATGGTGAGCGCGACAACTTCTGAAGTGCAACCCACCATGGGGGTCAAGATCTTCTCCGCGGGCGTGTCGGCCTGCCTGGCAGACATCATCACCTTCCC
GCTGGACACCGCCAAAGTCCGGCTTCAGATCCAAGGTGAGGGCCAGGCCTCCAGTACCATTAGGTATAAAGGTGTCCTAGGGACCATCACCACCCTG
GCAAAAACAGAAGGGTTGCCGAAACTGTACAGCGGTCTGCCTGCGGGCATTCAGAGGCAAATCAGCTTCGCCTCACTCAGGATCGGCCTCTATGATA

CTGTCCAAGAGTACTTCGCCTCAGAGAGAGAAAAGCCTGCCACGTTGGGAAACAAGATCTCAGCTGGCTTAATGACGGGAGGTGTGGCAGTGTTTAT
TGGGCAACCTACAGAGGTCGTGAAAGTCCGACTCCAAGCACAGAGCCATCTACATGGGATCAAACCCCGCTACACCGGGACCTACAACGCTTACAGA
ATCATAGCCACCACAGAGAGCTTGTCAACACTCTGGAAAGGGACAACCCCTAATCTAATGAGAAATATCATCATCAACTGTACAGAGCTGGTAACATAT
GACCTCATGAAGGGGGCCCTTGTGAACAACAAAATAATGGCAGATGAGGTCCCATGCCATTTGCTGTCAGCTCTTGTTGCCGGGTTTTGTACCACACT
CCTGGCCTCTCCGGTGGATGTGGTGAAGACGAGATTCATCAACTCTCTACCAGGACAGTACCCAAGTGTACCCAGCTGCGCAATGACCATGCTCACCA
AGGAAGGACCCACGGCCTTCTTCAAAGGGTTTGTGCCTTCTTTCCTGCGACTCGGGTCCTGGAATGTCATCATGTTTGTGTGCTTTGAACAGCTGAAA
AAGGAACTTACGAAGTCAAGGCAGCCGGTGGAATGCGCCACATAA

>*Acomys kempi*
ATGGTGAGCGCGCCAACTTCTGAAGTGCAACCCACCATGGGGGTCAAGATCTTCTCCGCGGGCGTGTCGGCCTGCCTGGCAGACATCATCACCTTCCC
GCTGGACACCGCCAAAGTCCGGCTTCAGATCCAAGGTGAGGGCCAGGCCTCCAGTACCATTAGGTATAAAGGTGTCCTAGGGACCATCACCACCCTG
GCAAAAACAGAAGGGTTGCCGAAACTGTACAGCGGTCTGCCTGCGGGCATTCAGAGGCAAATCAGCTTCGCCTCACTCAGGATCGGCCTCTATGATA
CTGTCCAAGAGTACTTCGCCTCAGAGAGAGAAAAGCCTGCCACGTTGGGAAACAAGATCTCAGCTGGCTTAATGACGGGAGGTGTGGCAGTGTTTAT
TGGGCAACCTACAGAGGTCGTGAAAGTCCGACTCCAAGCACAGAGCCATCTACATGGGATCAAACCCCGCTATACCGGGACCTACAACGCTTACAGA
ATCATAGCCACCACAGAGAGCTTGTCAACACTCTGGAAAGGGACAACCCCTAATCTAATGAGAAATATCATCATCAACTGTACAGAGCTGGTAACATAT
GACCTCATGAAGGGGGCCCTTGTGAACAACAAAATAATGGCAGATGAGGTCCCATGCCATTTGCTGTCAGCTCTTGTTGCTGGGTTTTGTACCACACT
CCTGGCCTCTCCGGTGGATGTGGTGAAGACGAGATTCATCAACTCTCTACCAGGACAGTACCCAAGTGTACCCAGCTGCGCAATGACCATGCTCACCA
AGGAAGGACCCACGGCCTTCTTCAAAGGGTTTGTGCCTTCTTTCCTGCGACTCGGGTCCTGGAATGTCATCATGTTTGTGTGCTTTGAACAGCTGAAA
AAGGAACTTACGAAGTCAAGGCAGACGGTGGAATGCACCACATAA

>*Peromyscus nudipes*
ATGGTGAGCCCGACAACTTCCGAAGTGCCTCCTACCATGGGGGTCAAGATCTTCTCAGCCGGCGTGGCCGCCTGTCTGGCAGATATCATCACCTTCCC
GCTGGACACAGCCAAAGTTCGACTTCAGATCCAAGGTGAAGGCCAGAAATCCAGTACCATTAGGTATAAAGGTGTCCTGGGGACCATCACCACCCTG
GCAAAAACAGAAGGGCTGCCAAAACTGTACAGCGGTCTGCCTGCTGGCATTCAGAGGCAAATCAGCTTTGCCTCACTCAGGATTGGCCTCTACGATA
CTGTCCAAGAGTACTTCTCTTCAGGGAGAGAAACACCTCCCACTTTGGGAAACAGGATCTCAGCTGGCTTGATGACTGGAGGTGTGGCAGTATTCATC
GGGCAACCTACAGAGGTCGTGAAAGTCAGACTCCAAGCACAGAGCCACCTACACGGGATCAAACCCCGCTACACTGGGACCTACAATGCTTACAGAA

TTATAGCTACAACAGAAAGCTTCTCAACACTCTGGAAAGGGACAACCCCTAATCTGATCAGGAATATCATCATCAATTGTACGGAGCTGGTAACATATG
ATCTCATGAAGGGGGCCCTTGTGAACAACCAAATACTGGCAGATGATGTTCCATGCCACTTACTGTCAGCTCTTGTCGCCGGGTTTTGCACCACACTCC
TGGCCTCTCCGGCGGATGTGGTAAAAACAAGATTCATCAACTCTCTGCCAGGACAGTACCCAAGTGTGCCCAGCTGTGCAATGACCATGTTCACCAAT
GAAGGACCAACAGCTTTCTTCAAAGGGTTTGTGCCCTCTTTCCTGCGACTCGCATCCTGGAATGTCATCATGTTCGTGTGCTTTGAACAGTTGAAGAA
AGAACTGATGAAGTCAAGGCAGACGGTGGACTGCTCCACATAA

>*Peromyscus polionotus*
ATGGTGAGCCCGACAACTTCCGAAGTGCCTCCTACCATGGGGGTCAAGATCTTCTCAGCCGGCTTGGCCGCCTGTCTGGCAGATATCATCACCTTCCCG
CTGGATACAGCCAAAGTCCGGCTTCAGATCCAAGGTGAAGGCCAGAACTCTAGTACCATTAGGTATAAAGGTGTCCTGGGGACCATCACCACCCTGGC
AAAAACAGAAGGGTTGCCGAAACTGTACAGTGGTCTGCCTGCTGGCATTCAGAGGCAAATCAGCTTTGCCTCACTCAGGATCGGCCTCTACGATACTG
TCCAAGAGTACTTCTCTTCAGGGAGAGAAACACCCCCCACTTTGATAAACAGGATCTCAGCTGGCTTGATGACTGGAGGTGTGGCAGTATTCATCGGG
CAACCTACAGAGGTCGTGAAAGTCAGACTCCAAGCACAGAGCCACCTGCATGGGATCAAACCCCGCTACACTGGGACCTACAATGCTTACAGAATTAT
AGCTACAACAGAAAGCTTCTCAACACTCTGGAAAGGGACAACCCCTAATCTGATCAGGAATATCATCATCAATTGTACAGAGCTGGTAACATATGACCT
CATGAAGGGGGCCCTTGTGAACAACCAAATTTTGGCAGATGACGTTCCCTGCCACTTACTGTCAGCTCTTGTCGCCGGGTTTTGCACCACACTCCTGG
CCTCTCCGGCGGATGTGGTAAAAACAAGATTCATCAACTCTCTGCCGGGACAGTACCCAAGTGTGCCCAGCTGTGCAATGACCATGTTCACCAACGAA
GGACCAACAGCTTTCTTCAAAGGGTTTGTGCCCTCTTTCCTGCGACTCGCATCCTGGAATGTCATCATGTTCGTGTGCTTTGAACAGCTGAAGAAAGA
ACTGATGAAGTCAAGGCAGACAGTGGACTGCTCCACATAA

>*Rhabdomys dilectus*
ATGGTGAACCCGACAACTTCCGAAGTGCAACCCACCATGGGGGTCAAGATCTTCTCCGCCGGCATTTCAGCCTGCTTGGCGGATATCATCACCTTCCC
GCTGGACACTGCCAAAGTCCGCCTTCAGGTCCAAGGTGAAGGCCCGGCTTCCAGCACCATTAGGTATAAAGGTGTCCTAGGGACCATCACCACCCTG
GCAAAAACAGAAGGATTTCCGAAACTGTACAGTGGCCTGCCCGCTGGCATTCAGAGGCAAATCAGCTTTGCCTCGCTCAGAATTGGCCTCTACGACA
CAGTCCAGGAGTACTTCTCTTCAGGGAGAGAAACACCTGCCTCTTTGGGGAACAAGATCTCAGCTGGCTTAATGACTGGAGGTGTGGCAGTATTCATT
GGGCAGCCTACAGAGGTTGTGAAGGTCAGAATGCAAGCACAGAGCCACCTGCACGGGATCAAACCCCGCTACACTGGGACCTACAATGCTTACAGA
GTTATAGCCACCACAGAAAGCTTGTCAACACTTTGGAAAGGGACGACCCCTAATCTAATGAGAAATGTCATCATCAATTGCACAGAGCTGGTAACATA
CGACCTCATGAAGGGGGCCCTTGTAAACAACAAAATAATGGCAGATGACGTCCCCTGCCATTTACTGTCAGCTCTTGTCGCTGGGTTTTGCACCACAC

TCCTGGCCTCTCCAGTGGATGTGGTAAAAACAAGATTCATCAACTCTCTGCCAGGACAGTATCCAAGCGTACCCAGCTGTGCAATGACCATGTACACC
AAGGAAGGACCGACAGCCTTTTTCAAAGGGTTTGTGCCTTCCTTTCTGCGACTCGGGTCCTGGAACGTCATCATGTTTGTGTGCTTTGAACAGCTGAA
AAAAGAGCTGACGAAATCCAGGCAGACTGTGGACTGCACCACATAA

>*Peromyscus aztecus*
ATGGTGAGCCCGACAACTTCCGAAGTGCCTCCTACCATGGGGGTCAAGATCTTCTCAGCCGGCGTGGCCGCCTGTCTGGCAGATATCATCACCTTCCC
ACTGGACACAGCCAAAGTTCGACTTCAGATCCAAGGTGAAGGCCAGAACTCCAGTACCATTAGGTATAAAGGTGTCCTGGGGACCATCACCACCCTG
GCAAAAACAGAAGGGCTGCCGAAACTGTACAGCGGTCTGCCTGCTGGCATTCAGAGGCAAATCAGCTTTGCCTCACTCAGGATTGGCCTCTACGATA
CTGTCCAAGAGTACTTCTCTTCAGGGAGAGAAACACCTCCCACTTTGGGAAACAGGATCTCAGCTGGCTTGATGACTGGAGGTGTGGCAGTATTCATC
GGGCAACCTACAGAGGTCGTGAAAGTCAGACTCCAAGCACAGAGCCACCTACACGGGATCAAACCCCGCTACACTGGGACCTACAATGCTTACAGAA
TTATAGCTACAACAGAAAGCTTCTCAACACTCTGGAAAGGGACAACCCCTAATCTGATCAGGAATATCATCATCAATTGTACAGAGCTGGTAACATATG
ACCTCATGAAGGGGGCCCTTGTGAACAACCACATACTTGCAGATGATGTTCCATGCCACTTACTGTCAGCTCTTGTCGCTGGGTTTTGTACCACACTCC
TGGCCTCTCCGGCGGATGTGGTAAAAACAAGATTCATCAACTCTCTACCAGGACAGTACCCAAGTGTGCCCAGCTGTGCAATGACCATGTTCACCAAC
GAAGGACCAACAGCTTTCTTCAAAGGGTTTGTGCCCTCTTTCCTGCGACTCGCATCCTGGAATGTCATCATGTTCGTGTGCTTTGAACAGCTGAAGAA
AGAACTGATGAAGTCAAGGCAGACGGTGGACTGCTCCACATAA

>*Mus spretus*
ATGGTGAGCCCAACAACTTCCGAAGTGCAACCCACCATGGGGGTCAAGATCTTCTCAGCTGGAGTTTCAGCTTGCCTGGCAGATATCATCACCTTCCC
GCTGGACACTGCCAAAGTCCGCCTTCAGATCCAAGGTGAAGGCCAGGCTTCCAGTACCATTAGGTATAAAGGTGTCCTAGGGACCATCACCaccctggcaaa
aacagaaggaTTGCCGAAACTGTACAGCGGTCTGCCTGCGGGCATTCAGAGGCAAATCAGCTTTGCCTCACTCAGGATTGGCCTCTACGACTCGGTCCAAG
AGTACTTCTCTTCAGGGAGAGAAACACCTGCCTCTCTCGGAAACAAGATCTCAGCCGGCTTAATGACTGGAGGTGTGGCAGTGTTCATTGGGCAGCCT
ACAGAGGTCGTGAAGGTCAGAATGCAAGCCCAGAGCCATCTGCATGGGATCAAACCCCGCTACACGGGGACCTACAATGCTTACAGAGTTATAGCCA
CCACAGAAAGCTTGTCAACACTTTGGAAAGGGACGACCCCTAATCTAATGAGAAATGTCATCATCAATTGTACAGAGCTGGTAACATATGACCTCATG
AAGGGGGCCCTTGTAAACAACAAAATACTGGCAGATGACGTCCCCTGCCATTTACTGTCAGCTCTTGTTGCCGGGTTTTGCACCACACTCCTGGCCTC
TCCAGTGGATGTGGTAAAAACAAGATTCATCAACTCTCTGCCAGGACAGTACCCAAGCGTACCAAGCTGTGCGATGTCCATGTACACCAAGGAAGGA
CCGACGGCCTTTTTCAAAGGGTTTGTGGCTTCTTTTCTGCGACTCGGGTCCTGGAACGTCATCATGTTTGTGTGCTTTGAACAGCTGAAAAAAGAGCT

GATGAAGTCCAGGCAGACAGTGGATTGTACCACATAA

>*Rhynchomys soricoides*
ATGGTGAGCCCGAAAACTTCCGAAGTGCAACCCACCATGGGGGTCAAGATCTTCTCAGCCGGCGTTTCAGCCTGCCTGGCAGACATCATCACCTTCCC
GCTGGATACTGCCAAAGTCCGCCTTCAGATCCAAGGTGAAGGCCAAGCTTCCAGTACCATTAGGTATAAAGGTGTCCTAGGGACCATCACCACCCTGG
CCAAAACAGAAGGATTGCCGAAACTGTACAGCGGTTTGCCTGCGGGCATTCAGAGGCAAATCAGCTTTGCCTCACTCAGGATTGGCCTCTACGATACG
GTCCAAGAGTACTTCTCTTCCGGAAGAGAAGGACCTGCCTCTTTGGGGAACAAGATCTCAGCTGGCTTAATGACTGGAGGTGTGGCAGTGTTCATTGG
GCAACCTACAGAGGTCGTGAAGGTCAGAATGCAAGCACAGAGCCATCTGCACGGGATCAAACCCCGCTACACTGGGACCTACAATGCTTACAGAGTT
ATAGCCACCACAGAGAGCTTGTCAACGCTTTGGAAAGGGACGACCCCGAATCTAATGAGAAATGTCATCATCAATTGTACAGAGCTGGTAACATATGA
CCTCATGAAGGGGGCCCTTGTCAACAACCAAATACTGGCAGATGACGTCCCCTGCCATTTACTGTCAGCTCTTGTCGCCGGGTTTTGCACCACACTCTT
GGCCTCTCCGGTGGATGTGGTAAAAACAAGATTCATCAACTCTCTACCAGGACAGTACCCAAGCGTACCCAGCTGTGCAATGACCATGTACACCAAGG
AAGGACCAATGGCCTTTTTCAAAGGGTTTGTGCCTTCTTTTCTGCGACTCGGGTCCTGGAACGTCATCATGTTTGTGTGCTTCGAACAGCTGAAAAAA
GAGCTGATGAAATCCAGGCAGACAGTGGACTGCACCACATAA

>*Sciurus vulgaris*
ATGGTGGGCCCCACCGCCTCCGACGTGCCCCCGACCATGGGCGTCAAGATCTTCTCGGCCGGAGTGGCAGCCTGCTTGGCGGATGTGATCACCTTCCC
GCTGGACACCGCCAAAGTCCGGCTACAGATCCAAGGAGAATGCCAGGTCTCCAGCGGTATTACTTATAAAGGTGTCCTGGGAACGATCACCACCCTGG
CGAAAACCGAAGGGCCCATGAAACTGTACAGCGGGCTGCCTGCTGGGCTGCAGAGGCAAATAAGCTTCGCCTCTCTCAGGATCGGACTCTACGATTC
GGTCCAGGAGTTCTTCACCTCAGGGAATGAAACAACGCCCAGTTTGGGAAGCAAGATCTCAGCCGGACTAACAACTGGAGGAGTAGCGGTGTTCATT
GGGCAGCCCACGGAGGTCGTGAAGGTCAGGCTGCAAGCACAGAGCCATCTACATGGCCTCAAACCTCGCTACACTGGGACTTACAACGCATACAGAA
TTATAGCGACAACAGAAAGCTTGAGGAGTCTCTGGAAAGGGACTACTCCTAATCTGTTGAGAAACATCATCATCAATTGCACAGAGCTAGTAACATAC
GACCTGATGAAGGGGGCCCTTGTGAGAAACAAACTACTAGCAGATGACGTCCCCTGTCACCTGCTGTCTGCTCTCATTGCTGGATTTTGCACCACGCT
TCTGTCCTCCCCGGTGGATGTGGTGAAGACCAGATTTATTAACTCTCCACCAGGGCAGTATACCAGTGTGCCCAGCTGCGCAATGACAATGCTCCACA
AGGAAGGGCCGACGGCGTTTTTCAAAGGATTTGTACCTTCCTTCCTGCGACTGGGATCCTGGAATATCATTATGTTCGTGTGCTTTGAACAGCTGAAAC
GAGAATTGATGAAGTCTAGGCAGACTGTGGACTGTGCCACATGA

>*Cricetomys gambianus*
ATGGTGAGCCCTGCAACTTCCGAAGTGCCCCCCACCATGGGGGTCAAGATCTTCTCAGCCGGCGTGGCCGCCTGCCTGGCAGACATCATCACCTTCCC
GCTGGACACCGCCAAAGTCCGGCTTCAGATCCAAGGTGAAGGTCAGGCCTCCAGTACCATTCGGTACAAGGGTGTGCTGGGAACCATCACCACCCTG
GCAAAGACAGAAGGGGTGCCAAAACTCTACAGTGGGCTACCTGCTGGCATTCAGAGGCAAATCAGCTTCGCCTCGCTCAGGATTGGCCTCTACGACA
CTGTCCAAGACTACTTCTCTTCAGGGAGAGACACACCTGCCACTTTGGGAAACAAGATCTCAGCTGGCTTAATGACTGGAGGCGTAGCAGTGTTCATC
GGGCAACCTACAGAGGTCGTGAAAGTCAGACTCCAAGCACAGAGCCACCTACATGGGATCAAACCCCGCTACACGGGGACCTACAATGCTTACAGAA
TTATAGCCACAACAGAAAGCCTGTCAACACTCTGGAAAGggACAACCCCTAATCTAATGAGGAATGTCATCATCAATTGTACAGAACTGGTAACATATG
ACCTCATGAAGGGGGCCCTTGTGAACAACCAAATACTAGCAGatgacgtCCCATGCCACTTACTGTCAGCTCTCGTTGCCGGGTTTTGCACCACACTCCTG
GCCTCTCCCGTGGATGTGGTAAAAACAAGATTCATCAACTCTGTACCAGGCCAGTACCCAAGCGTACCCAGCTGTGCGATGACCATGTTCACCAAGGA
AGGACCATCCGCCTTTTTCAAAGGATTGATGCCTTCTTTCCTGCGACTCGGGTCCTGGAACGTCATCATGTTTGTGTGCTTTGAACAGCTGAAAAAGG
AACTGATGAAGTCCAGGCAGACAGCGGGCTGCACCACATAA

>*Psammomys obesus*
ATGGTGAGCCCAACAACTTCTGAAGTGCAGCCCACCATGGGGGTCAAGATCTTCTCAGCCGGTGTGGCGGCCTGCCTGGCAGATATCATTACCTTCCC
GCTGGACACCGCCAAAGTCCGGCTTCAGATCCAAGGTGAAGGCCCAGCCTCCAGCACCATTCGGTATAAAGGTGTCCTAGGGACCATCACCACCCTG
GCAAAAACAGAAGGGGTGCCGAAACTGTACAGCGGTCTGCCTGCGGGCATTCAGAGGCAAATCAGCTTCGCCTCACTCAGGATTGGCCTCTACGATA
CCGTCCAAGAGTACTTCGCTTCAGGGAGAGAAACACCTGCCACTTTGGGGAACAAGATCTCAGCTGGCTTAATGACTGGAGGTGTGGCAGTATTCATC
GGGCAACCTACAGAAGTCGTGAAAGTCAGAATGCAAGCACAGAGCCATCTACACGGCATCAAACCCCGCTACACTGGAACTTACAATGCTTACAGAA
TTATAGCCACAACAGAAAGCTTGTCAACACTCTGGAAAGGGACAACCCCTAATCTAATGAGAAATGTCATCATCAATTGTACAGAGCTGGTAACATATG
ACCTCATGAAGGGGGCCCTGGTGAATAACCGAATACTGGCAGATGACGTCCCATGCCATTTGCTGTCAGCTCTTGTTGCCGGCTTCTGCACCACACTG
CTGGCCTCTCCAGTGGATGTGGTGAAAACGAGATTCATCAACTCTCTACCAGGACAGTACCCGAGTGTGCCCAGCTGTGCAATGACCATGTTCAACAA
GGAAGGACCAACGGCCTTCTTCAAAGGGTTTGTGCCTTCTTTCCTGCGACTGGGCTCCTGGAACGTCATCATGTTTGTGTGCTTTGAGCAGCTGAAAA
AAGAACTGATGAAGTCAAGGCAGACGGTGGACTGTACCACATAA

>*Sigmodon hispidus*
ATGGTGAGCCAAACAACTTCCGAAGTGCACCCCACCATGGGGGTCAAGATCTTCTCAGCAGGTGTGGCAGCCTGCCTGGCAGATATCATCACCTTCCC

ACTGGACACAGCCAAAGTCCGGCTTCAGATCCAAGGTGAAGGCCAGAATTCTAGCACCATTAGGTATAAAGGTGTCCTGGGGACCATCACCACCCTG
GCAAAAACAGAAGGGTTGCCGAAACTGTATAGCGGTCTGCCCGCTGGCATTCAGAGGCAAATCAGCTTTGCCTCACTCAGGATTGGCCTCTACGATAC
TGTGCAAGAGTACTTCTCTTCAGGGAGAGAAACACCTCCCACTTTGGCAAACAGGATCTCTGCAGGCTTAATGACTGGAGGTGTGGCAGTATTCATCG
GGCAACCCACTGAGGTCGTAAAAGTCAGACTCCAAGCACAAAGCCACCTACATGGGATCAAACCCCGCTACACTGGGACCTACAATGCCTACAGAAT
TATAGCCACAACAGAAAGCTTATCTACACTCTGGAAAGGGACAACCCCTAATCTGATGAGGAATGTCATCATCAATTGTACAGAGCTGGTAACATATGA
CCTCATGAAAGGGGCTCTTGTGAACAACAAAATAATGGCAGATGATGTCCCATGCCATTTACTGTCAGCCTTTGTCGCCGGGTTTTGTACCACACTCCT
GGCCTCTCCAGCAGATGTGGTGAAAACAAGATTCATCAACTCTCTACCAGGACAGTATCCAAGTGTACCCAACTGCGCAATGACCATGTTAACCAAGG
AAGGACCATCGGCTTTTTTCAAAGGGTTTGTGCCTTCTTTCCTGCGACTTGCATCCTGGAATGTCATCATGTTTGTGTGCTTTGAACAGCTGAAAAAAG
AATTGATGAAGTCAAGGCAGACAGTGGACTGCACCACATAA

>*Lophiomys imhausi*
ATGGTGAGCCCGACAACTTCTGAAGTGCAGCCCACCATAGGGGTCAAGATCTTCTCAGCCGGCGTGGCGGCCTGCCTGGCAGATATCATCACCTTCCC
ACTGGACACCACCAAAGTCCGGCTTCAGATCCAAGGTGAAGGCCAGGTTTCCAGTATCATTAGATATAAAGGTGTCCTAGGAACCATGTCCACCCTGG
CAAAAACAGAAGGGTGGCCGAAACTGTACAGCGGTCTGCCTGCGGGGATTCAGAGGCAAATCAGCTTCGCCTCACTCAGGATTGGCCTCTACGACAC
TGTCCAAGAGTACTTCTCGTCAGGGAGAGAAACACCTGCCACTTTGGGGAACAAGATCTCAGCTGGCTTAATGACTGGAGGTGTGGCAGTGTTCATC
GGGCAACCTACAGAGGTCGTGAAAGTCAGACTCCAAGCACAGAGCCACCTACATGGGATCAAACCCCGCTACACTGGAACCTACAATGCTTACAGAA
TTATAGCCACAACAGAAAGCTTGTCAACACTCTGGAAAGGGACGACTCCTAATCTAATGAGAAATATCATCATCAATTGTGCAGAGCTGGTAACTTATG
ACCTTATGAAGGGGGCCCTTGTGAAGAACCAAATACTGGCAGATGACGTCCCATGCCATTTACTGTCAGCCCTTGTTGCCGGGTTTTGCACCACATTTC
TGGCCTCTCCGGTGGATGTGGTAAAAACGAGATTCATCAACTCTCTACCAGGACAGTACCCAAGTGTACCAAGCTGTGCGATGACCATGTTTACCAAG
GAAGGACCAACAGCCTTTTTCAAAGGGTTGGTGCCTTCTTTCCTACGACTCGGGTCCTGGAACGTCATCATGTTTGTGTGCTTTGAACAGCTGAAGAA
GGAACTGACCGACCCAAGGCAGATGGTGGACTGCACCACATGA

>*Acomys russatus*
ATGGTGAGCGCGACAACTTCTGAAGTGCAACCCACCATGGGGGTCAAGATCTTCTCCGCGGGCGTGTCGGCCTGCCTGGCAGACATCATCACCTTCCC
GCTGGACACCGCCAAAGTCCGGCTTCAGATCCAAGGTGAAGGCCAGGCCTCCAGTACCATTAGGTATAAAGGTGTCCTAGGGACCATCACCACCCTG
GCAAAAACAGAAGGGTTGCCGAAACTGTACAGCGGTCTGCCTGCGGGCATTCAGAGGCAAATCAGCTTCGCCTCACTCAGGATCGGCCTCTATGATA

CTGTCCAAGAGTATTTCGcctcagagagagaaaAGCCTGCCACTTTGGGAAACAAGATCTCAGCTGGCTTAATGACTGGAGGTGTGGCAGTGTTTATTGGGCA
ACCTACAGAGGTCGTGAAAGTCCGACTCCAAGCACAGAGCCATCTACATGGGATCAAACCCCGCTACACCGGGACCTACAACGCTTACAGAATCATA
GCCACCACAGAAAGCTTGTCAACACTCTGGAAAGGGACAACCCCTAATCTAATGAGAAATGTCATCATCAACTGTACAGAGCTGGTAACATATGACCT
CATGAAGGGGGCCCTTGTGAACAACAAAATAATGGCAGATGAGGTTCCATGCCATTTGCTGTCAGCTCTTGTTGCCGGGTTTTGTACTACACTCCTGGC
CTCTCCAGTGGATGTGGTGAAGACGAGGTTCATCAACTCTCTACCAGGACAGTACCCAAGTGTACCCAACTGCGCAATGACCATGCTCACCAAGGAA
GGACCCACAGCCTTCTTCAAAGGGTTTGTGCCTTCTTTCCTGCGACTCGGGTCCTGGAATGTCATCATGTTTGTGTGCTTTGAACAGCTGAAAAAGGA
ACTAACGAAGTCAAGGCAGACAGTGGAATGCACCACATAA

>*Phodopus roborovskii*
ATGGTGAGCCAGACAACTTCCGAAGTGCACCCCACCATGGGGGTCAAGATCTTCTCAGCCGGTGTGGCGGCCTGCCTGGCAGATATCATCACCTTCCC
GCTGGACACGGCCAAAGTCCGGCTTCAGATCCAAGGCGAAGGCCAGACCTCCAGTACCATTAGGTATAAAGGCGTGCTTGGGACCATCACCACCCTA
GCAAAAACAGAAGGGCTGCCGAAACTGTACAGTGGTCTGCCTGCTGGCATCCAGAGACAAATCAGCTTTGCCTCTCTCAGGATTGGCCTCTACGATAC
TGTCCAAGAGTACTTCTCTTCAGGGAAAGAAACTCCTCCCACTTTGGGAAACAGGATCTCAGCTGGCTTAATGACTGGAGGTGTGGCAGTATTCATCG
GGCAACCTACTGAGGTTGTGAAAGTCAGACTCCAGGCACAGAGCCACCTACATGGGATCAAACCCCGCTACACGGGTACCTACAATGCTTACAGAATT
ATAGCCACAACAGAAAGCCTGTCAACACTCTGGAAAGGAACGACCCCTAATCTGTTGAGGAATGTCATCATCAACTGTACAGAGCTGGTAACATATGA
CCTCATGAAGGGGGCCCTTGTGAACAACCAAATACTGGCAGATGACGTGCCATGCCACTTACTGTCAGCTCTTGTCGCCGGCTTTTGCACCACACTCC
TGGCCTCTCCAGCAGATGTGGTAAAAACAAGATTCATCAACTCTCTACCAGGACAGTACCCAAGTGTCCCCAGTTGCGCAATGACCATGTTCACCAAG
GAAGGACCAACAGCTTTTTTCAAAGGGTTTGTGCCTTCTTTCCTGCGACTGGCATCCTGGAATGTCATCATGTTTGTGTGCTTTGAACAGCTGAAGAA
AGAGTTGATGAAGTCGAGACAGACGGTGGACTGCACCACATAA

>*Microtus arvalis*
ATGGTGAGCCCGACAACTTCCGAAGTGCACCCCACCATGGGGGTCAAGATCTTCTCAGCTGGCATATCCGCCTGCCTGGCAGATATCATCACCTTCCCA
CTGGACACAGCCAAAGTCCGGCTTCAGATCCAAGGTGAAGGCCAGACCTCCACTACCATTAGGTATAAAGGTGTCCTGGGAACCATCACCACCCTGG
CAAAAACAGAAGGGTTGCCGAAACTGTACAGCGGTCTGCCTGCTGGCATTCAGAGGCAAATCAGCTTCGCCTCACTCAGGATCGGTCTCTATGATACT
GTCCAAGAGTACTTCTCTTCAGGGAAAGAAACGCCTCCCACTTTGGGAAACAGGATCTCAGCCGGCTTATTGACTGGAGGTGTGGCAGTATTCATCGG
GCAACCTACCGAGGTCGTGAAAGTCAGACTCCAAGCACAGAGTCACTTACACGGGATCAAACCCCGCTACACAGGGACCTACAATGCTTACAGAATT

ATAGCCACAACAGAAAGCTTGTCAACCCTCTGGAAAGGGACAACCCCTAATCTGATGAGAAATGTCATCATCAATTGTACAGAGCTGGTAACATATGA
CCTCATGAAGGGGGCCCTTGTGAACAACCAAATACTGGCAGACGACGTGCCCTGCCATTTACTCTCAGCTCTTGTGGCTGGGTTTTGCACCACCTTCC
TGGCCTCTCCGGCAGATGTGGTAAAAACAAGATTCATCAACTCTCTACCAGGACAGTACCCGAGTGTACCCAGCTGTGCAATGGCCATGCTCACCAAG
GAAGGACCAACGGCTTTCTTCAAAGGGTTTGTGCCTTCTTTCCTGCGACTGGCGTCCTGGAATGTCATCATGTTTGTGTGCTTCGAACAGCTGAAGAA
GGAACTGATGAAGTCCAGGCAGACGGTGGACTGCACCACATAA

>*Ondatra zibethicus*
ATGGTGAACCCGACAACTTCCGAAGTGCACCCCACCATGGGGGTCAAGATATTCTCAGCTGGCATATCCGCCTGCCTGGCAGATATCATCACCTTCCCA
CTGGACACAGCCAAAGTCCGGCTTCAGATCCAAGGTGAAGGCCAGACCTCCAGCACCATTAGGTATAAAGGTGTCCTGGGAACCATCACCACCCTGG
CAAAAACAGAAGGGTTGCCGAAACTGTACAGCGGTCTGCCTGCTGGCATTCAGAGGCAAATCAGCtttgcctcactcaggattgGTCTCTATGATACTGTCCAAG
AGTACTTCTCTTCAGGGAAAGAAACGCCTCCCACTTTGGGAAACAGGATCTCAGCTGGATTAATGACTGGAGGTGTGGCAGTATTCATTGGGCAACCT
ACTGAGGTCGTGAAAGTCAGACTCCAGGCACAGAGCCACTTACATGGGATCAAACCCCGCTACACAGGGACCTACAATGCTTACAGAATTATAGCCA
CAACAGAAAGCTTCTCAACACTCTGGAAAGGGACGACCCCTAATCTCATGAGAAATGTCATTATCAATTGTACAGAGCTGGTAACATATGACCTCATGA
AGGGGGCCCTTGTGAACAACCAAATACTGGCAGACGACATGCCGTGCCATTTGCTGTCAGCTCTTGTCGCCGGGTTTTGCACCACCCTTCTGGCCTCT
CCGGCGGATGTGGTAAAAACAAGATTCATCAACTCTCTACCAGGACAGTACCCAAGTGTACCCAGCTGTGCAATGACCATGCTCACCAAGGAAGGAC
CAACGGCTTTTTTCAAAGGGTTTGTGCCTTCTTTCCTGCGACTGGCGTCCTGGAATGTCATCATGTTTGTGTGCTTCGAACAGCTGAAGAAGGAATTGA
TGAAGTCAAGGCAGACGGTGGACTGCACCACATAA

>*Ellobius lutescens*
ATGGTGAACCCGACAACTTCCGAAGTGCACCCCACCATGGGGGTTAAGATCTTCTCAGCTGGCATATCCGCCTGCCTGGCAGATATCATCACCTTCCCA
CTGGACACAGCCAAAGTCCGGCTTCAGATTCAAGGTGAAGGCCAGACCTCCAGTACCATTAGGTATAAAGGTGTCCTGGGAACCATCACCACCCTGG
CAAAAACAGAGGGGTTGCCGAAACTGTACAGCGGTCTGCCTGCTGGCATTCAGAGGCAAATCAGCTTCGCCTCACTCAGAATTGGTCTCTATGATACT
GTCCAAGAGTACTTCTCTTCagggaaagaaaCGCCTCCCACTTTGGGAAACAGGATCTCGGCTGGCTTAATGACTGGAGGTGTGGCAGTATTCATCGGGCAA
CCTACCGAGGTCGTGAAAGTCAGACTCCAAGCACAGAGCCACTTACATGGGATCAAACCCCGCTACACAGGGACCTACAATGCTTACAGAATTATAGC
CACAACAGAAAGCTTGTCAACCCTCTGGAAAGGGACGACCCCTAATCTGATGAGAAATGTCATTATCAATTGTACAGAGCTGGTAACATATGACCTCAT
GAAGGGGGCCCTTGTGAACAACCAAATACTGGCAGACGATGTGCCCTGCCATTTACTGTCAGCTCTTGTGGCCGGGTTTTGCACCACCTTCCTGGCCT

CTCCAGCAGATGTGGTAAAAACAAGATTCATCAACTCTCTACCAGGACAGTACCCAAGTGTACCCAGCTGTGCAATGACCATGCTCACCAAGGAAGG
ACCAATGGCTTTTTTCAAAGGGTTTGTGCCTTCTTTCCTGCGGCTGGCGTCCTGGAATGTCATCATGTTTGTGTGCTTCGAACAGCTGAAGAAGGAATT
GATGAAGTCAAGGCAGACGGTGGACTGTACCACATAA

>*Hylomyscus alleni*
ATGGTGAACCCGACAACTTCCGAAGTGCAACCCACCATGGGGGTCAAGATCTTCTCAGCCGGCGTTTCAGCCTGCCTGGCAGATATCATCACCTTCCC
GCTGGACACCGCCAAAGTCCGCCTTCAGATCCAAGGTGAAGGCCAGGCTTCCAGTACCATTAGGTATAAAGGTGTCCTAGGGACCATCACCACCCTGG
CAAAAACAGAAGGATTGCCGAAACTGTACAGCGGTCTGCCTGCAGGCATTCAGAGGCAAATCAGCTTTGCCTCACTCAGGATTGGCCTCTACGATAC
GGTCCAAGAGTACTTCTCCTCGGGGAGAGAAACACCCGCCTCTTTAGGGAACAAGATCTCAGCTGGCTTAATGACGGGAGGTGTGGCCGTGTTCATT
GGGCAGCCCACAGAGGTCGTGAAGGTCAGGATGCAAGCACAGAGCCATCTGCATGGGATCAAACCCCGCTACACTGGGACCTACAATGCTTACAGAG
TTATAGCCACCACAGAAAGCTTGTCAACACTTTGGAAAGGGACGACCCCTAATCTAATGAGAAATGTCATCATCAATTGCACAGAGCTGGTAACATATG
ACCTTATGAAGGGGGCCCTTGTAAACAACAAAATACTGGCAGATGACGTCCCCTGCCATTTACTGTCAGCTCTCGTCGCTGGGTTTTGTACCACACTCC
TGGCCTCTCCGGTGGATGTGGTAAAAACAAGATTCATCAACTCTCTGCCCGGACAGTATCCAAGTGTACCCAGCTGTGCAATGACCATGTACACCAAG
GAGGGACCGACGGCCTTTTTCAAAGGGTTTGTGCCTTCTTTTCTGCGACTCGGCTCCTGGAACGTCATCATGTTTGTATGCTTTGAACAGCTGAAAAA
AGAGCTGACGAAGTCCAGGCAGACAGTGGATTGTACCACATAA

>*Mus minutoides*
ATGGTGAGCCCAACAACTTCCGAAGTGCAACCCACCATGGGGGTCAAGATCTTCTCAGCCGGAGTTTCAGCCTGCCTAGCAGATATCATCACCTTCCC
ACTGGACACTGCCAAAGTCCGCCTTCAGATCCAAGGTGAAGGCCAGGCTTCCAGTACCATTAGGTATAAAGGTGTCCTAGGGACCATCACCACCCTGG
CAAAAACAGAAGGACTGCCGAAACTGTACAGCGGTCTGCCTGCAGGCATTCAAAGGCAAATCAGCTTTGCCTCGCTCAGGATTGGCCTCTACGACTC
CGTCCAAGAGTACTTCTCTTCAGCGAGAGAAACACCTGCCTCTCTCGGAAACAAGATCTCAGCTGGCTTAATGACTGGAGGTGTGGCGGTGTTCATTG
GGCAGCCTACAGAGGTCGTGAAGGTCAGAATGCAAGCCCAGAGCCATCTGCATGGGATCAAACCCCGCTACACGGGGACCTACAATGCTTACAGAGT
TATAGCCACCACAGAAAGCTTGTCAACGCTTTGGAAAGGGACAACCCCTAATCTAATGAGAAATGTCATCATCAATTGTACAGAGCTGGTAACATATGA
CCTCATGAAGGGGGCCCTTGTAAACAACAAAATACTGGCAGATGACGTCCCCTGCCATTTACTGTCAGCTCTTGTTGCCGGGTTTTGCACCACACTCCT
GGCCTCTCCAGTGGATGTGGTAAAAACAAGATTCATCAACTCTCTGCCAGGACAGTACGCAAGCGTGCCAAGCTGTGCGATGACCATGTACACCAAG
GAAGGACCAACGGCCTTTTTCAAAGGGTTTTTGGCTTCTTTTCTGCGACTCGGGTCCTGGAACGTCATCATGTTTGTGTGCTTTGAACAGCTGAAAAA

AGAGCTGATGAAGTCCAGGCAGACAGTGGATTGCACCACATAA

>*Ellobius talpinus*
ATGGTGAACCCGACAACTTCCGAAGTGCACCCCACCATGGGGGTCAAGATCTTCTCAGCTGGCATATCCGCCTGCCTGGCAGATATCATCACCTTCCCA
CTGGACACAGCCAAAGTCCGGCTTCAGATCCAAGGTGAAGGCCAGACCTCTAGTACCATTAGGTATAAAGGTGTCCTGGGAACCATCACCACCCTGG
CAAAAACAGAAGGGTTGCCGAAACTGTACAGCGGTCTGCCTGCTGGCATTCAGAGGCAAATCAGCTTCGCCTCACTCAGGATTGGTCTCTATGATACT
GTCCAAGAGTACTTCTCTTCAGGGAAAGAAACGCCTCCCACTTTGGGAAACAGGATCTCAGCTGGCTTAATGACTGGAGGTGTGGCAGTATTCATTGG
GCAACCTACCGAGGTCGTGAAAGTCAGACTCCAAGCACAGAGCCACTTACATGGGATCAAACCCCGCTACACAGGGACCTACAATGCTTACAGAATT
ATAGCTACAACAGAAAGCTTGTCAACCCTCTGGAAAGGGACGACCCCTAATCTGATGAGAAATGTCATTATCAATTGTACAGAGCTGGTAACATATGAC
CTCATGAAGGGGGCCCTTGTGAACAACCAAATACTGGCAGACGATGTGCCCTGCCATTTACTGTCAGCTCTTGTGGCCGGGTTTTGCACCACCTTCCT
GGCCTCTCCAGCAGATGTGGTAAAAACAAGATTCATCAACTCTCTACCAGGACAGTACCCAAGTGTACCCAGCTGTGCAATGACCATGCTCACCAAGG
AAGGACCAACGGCTTTTTTCAAAGGGTTTGTGCCTTCTTTCCTGCGGCTGGCGTCGTGGAATGTCATCATGTTTGTGTGCTTCGAACAGCTGAAGAAG
GAATTGATGAAGTCAAGGCAGACGGTGGACTGCACCACATAA

>*Marmota marmota*
ATGGTGAGCCCCACAGCCTCCGACGTGCACCCGACCATGGGCATCAAGATCGTCTCAGCCGGAGTGTCAGCCTGCTTGGCGGATGTGATCACCTTCCC
GCTGGACACCGCCAAAGTCCGGCTACAGATCCAAGGCGAATTCCCAGTCTCCAGTGGTATTAAGTATAAAGGTGTCCTGGGAACAATCACCACCCTGG
CAAAAACCGAAGGGCCCATGAAACTGTACAGTGGGTTGCCTGCGGGATTGCAAAGGCAAATAAGCTTCGCCTCTCTTAGGATCGGCCTCTATGATTCT
GTCCAGGAGTTCTTCACCTCAGGGAATGAAACAACACCCAGTTTGGGAAGCAAAATCTCAGCCGGACTCACAACTGGAGGAGTGGCAGTGTTCATTG
GGCAGCCCACCGAGGTCGTGAAGGTCAGGCTCCAAGCACAGAGCCACCTACACGGGCTCAAACCTCGCTACACTGGGACCTACAACGCGTACAGAA
TTATAGCAACAACAGAGAGCTTCAGGAGTCTTTGGAAAGGGACTACTCCTAATCTGTTGAGAAATGTCATCATCAATTGCACAGAGTTGGTAACATATG
ACCTGATGAAGGGGGCTCTTGTGAGAAACAAAATCCTAGCAGATGATGTCCCCTGCCACCTGCTGTCCGCTTTCGTCGCTGGATTTTGCACCACACTT
CTGTCCTCGCCGGCTGATGTGGTGAAGACCAGATTTATTAACTCTCCGCCAGGGCAGTATACCAGCGTGCCCAGATGTGCAATGACAATGCTCCACCA
GGAAGGGCCGTCGGCGTTTTTCAAAGGATTTGTACCTTCCTTCCTGCGACTGGCATCCTGGAACGTCATTATGTTCGTGAGCTTTGAACAGCTGAAAC
GAGAATTGATGAAGTCCAGGCAGACAGTGGACTGCGCCACGTGA

>*Castor canadensis*
ATGGTGAGCGCCACAGCCTCGGAAGTGCCCCCGACCACGGGGGTCAAGATCTTCTCGGCCGGAGTGGCCGCCTGCCTGGCGGATGTGATCACCTTCC
CACTGGACACCGCCAAAGTCAGGCTACAGATACAAGGGGAAAGCCAGATATCCAGTGGCATTAGGTACAAAGGCGTCCTGGGAACCATCACCACTCT
GGCGAAAACAGAAGGGTCTATGAAGCTGTACAGCGGGCTGCCTGCTGGCATCCAGAGGCAAATAAGCTTCGCCTCCCTCAGGATCGGCCTGTATGAC
ACAGTCCAGGAGTACTTCACATCAGAGGAAGGAAAGGCACCTAGCTTGGGAAACAAGATTGCTGCTGGCTTAATGACCGGAGGCGTGGCAGTGTTCA
TCGGGCAACCCACGGAGGTGGTGAAAGTCAGGCTGCAAGCACAGAGCCATCTACATGGTCTGAAACCTCGCTACACTGGAACCTACAATGCTTACAG
AATTATAGCCACAACAGAAAGCTTGACAACTCTTTGGAAAGGCACTACTCCTAACCTACTGAGAAATATCATCATCAATTGTACAGAGCTAGTGACATA
TGACCTGATGAAGGGGTtctttgtgaaaaacaaaatactagcaGATGACGTCCCCTGTCACTTACTGTCGGCTCTTATTGCTGGGTTTTGCACAACACTTCTGTCCTCT
CCTGTGGATGTGGTAAAAACCAGATTTATTAATTCTCCAGCTGGACAGTACATAAGCGTGCCTAGCTGTGCAATGACAATGTTCACTAAGGAAGGACCA
ACGGCTTTTTTCAAAGGATTCATGCCTTCCTTCCTACGACTTGGATCCTGGAACGTCATCATGTTTGTGTGCTTTGAACAGCTGAAACGGGAATTGATG
AAGTCGAGGCAGACTGCGGGTTATGCCACGTAG

>*Cavia aperea*
ATGGTGAGCCCGACGGTGTCAGACGTGCACCCCACCATGGGGGTCAAGATCTTCTCAGCCGGAGTGTCGGCCTGCTTGGCGGATGTGATCACCTTCCC
GCTGGACACCGCCAAAGTCCGCCTACAGATCCAAGGCGAAAGCCCGACCTCCAGCGGCATTCGGTATAAGGGCGTCCTGGGGACTATCACCACGCTG
GCAAAGACTGAAGGGCCCGTGAAACTGTACAGCGGGCTCCCCGCAGGCCTGCAGAGGCAGATCAGCTTCGCCTCCCTCAGGATCGGCCTGTATGACA
CCGTCCAGGAGTTCTACACCTCGGAGAAAGATGCAACGCCTAGTTTGGGAAGCAGGATTGCAGCTGGCTTAACCACCGGAGGAGTGGCAGTATTCAT
CGGGCAGCCCACGGAGGTCGTGAAGGTCCGACTCCAGGCGCAGAGCCATCTACATGGCCTCAAACCTCGCTACACTGGGACTTACAATGCTTACAGA
ATTATAGCAACAACAGAAAGCTTGAAAAGTCTTTGGAAAGGGACAACTCCCAATCTGTTGAGAAATATCATCATCAACTGCACAGAGTTAGTCACGTA
CGACCTGATGAAGGGCGCCCTTGTGAGAAACAAGATACTAGCAGATGACGTCCCCTGCCATTTGTTGTCAGCTCTTATTGCTGGGTTTTGCACAACGC
TTCTGTCCTCTCCAGTGGATGTGGTGAAAACCAGGTTTATTAATTCTCCACCGGGACAGTACCTAAGTGTGCCCAGCTGTGCAATGACAATGCTCCACA
AGGAAGGACCCACGGCTTTTTTCAAAGGATTCGTACCTTCCTTCCTGCGACTGGCATCCTGGAACGTCATTATGTTTGTGTGCTTTGAGCAGCTGAAGA
AGGAGTTGATGAAGTCAAGGCAGCCCGTGGACTACGCCACTTAA

>*Cricetulus griseus*
ATGGTGAGCCCCACAACTTCCGAAGTGCACCCCACCATGGGGGTCAAGATCTTCTCAGCTGGCGTGGCGGCCTGCCTGGCAGATATCATCACCTTCCC

GCTGGACACAGCCAAAGTCCGGCTTCAGATCCAAGGTGAAGGTCAGACCTCCAGTACCATTAGGTACAAAGGTGTCCTGGGGACTATCACCACCCTG
GCAAAAACAGAAGGGTTGCCGAAACTGTACAGTGGTCTGCCTGCTGGCATCCAGAGACAAATCAGCTTCGCATCACTCAGGATTGGCCTCTATGATAC
TGTCCAAGAGTACTTCTCTTCAGGGAAAGAAACACCTCCCACTTTGGGAAATAGGATCTCAGCTGGTTTAATGACTGGAGGTGTGGCAGTATTCATCG
GGCAACCTACCGAGGTCGTGAAAGTCAGACTCCAAGCACAGAGCCACCTACATGGGATCAAACCCCGCTACACTGGGACCTACAATGCTTACAGAAT
TATAGCCACAACAGAAAGCTTGTCAACACTCTGGAAAGGGACGACCCCTAATCTGTTGAGGAATGTCATCATCAACTGTACAGAGTTGGTAACATATG
ACCTCATGAAGGGGGCCCTTGTGAACAACCAAATACTGGCAGATGATGTGCCATGCCATTTACTGTCAGCTCTTGTCGCTGGGTTTTGCACCACATTCC
TGGCCTCTCCGGCAGATGTGGTAAAAACAAGATTCATCAACTCTCTACCAGGACAGTACCCAAGTGTCCCCAGCTGCGCAATGACCATGTTCACCAAG
GAAGGACCAACAGCTTTTTTCAAAGGGTTTGTGCCTTCTTTCCTGCGACTGGCATCGTGGAATGTCATCATGTTTGTGTGCTTTGAACAGCTGAAGAA
AGAACTGATGAAGTCGAGGCAGACAGTGGACTGCACCACATAA

>*Arvicola amphibius*
ATGGTGAGCCCGACAACTTCCGAAGTGCACCCCACCATGGGGGTCAAGATCTTCTCAGCTGGCATATCCGCCTGCCTGGCAGATATCATCACCTTCCCA
CTGGACACAGCCAAAGTCCGGCTTCAGATCCAAGGTGAAGGCCAGACCTCCAGTATCATTAGGTATAAAGGTGTCCTGGGAACCATCACCACCCTGG
CAAAAACAGAAGGGTGGCAGAAACTGTACAGCGGTCTGCCTGCTGGCATTCAGAGGCAAATCAGCTTCGCCTCACTCAGGATTGGGCTCTATGATAC
TGTCCAAGAATACTTCTCTTCAGGGAAAGAAACGCCTCCCACTTTGGGAAACAGGATCTCAGCTGGCTTAATGACTGGAGGTGTGGCAGTATTCATCG
GGCAACCTACCGAGGTCGTGAAAGTCAGACTCCAAGCACAGAGCCACTTACACGGGATCAAACCCCGCTACACAGGGACCTACAATGCTTACAGAAT
TATAGCCACAACAGAAAGCTTGTCAACCCTCTGGAAAGGGACGACCCCAAATCTGATGAGAAATGTCATTATCAATTGTACAGAGCTGGTAACATATG
ACCTCATGAAGGGGGCTCTTGTGAACAACCAAATACTGGCAGACGACGTGCCCTGCCATTTACTGTCAGCTCTTGTGGCCGGGTTTTGCACCACCTTC
CTGGCCTCTCCAGCGGATGTGGTAAAAACAAGATTCATCAACTCTCTACCAGGACAGTACCCGAGTGTACCCAGCTGTGCAATGGCCATGCTCACCAA
GGAAGGACCAACGGCTTTTTTCAAAGGGTTTGTGCCTTCTTTCCTGCGACTGGCATCCTGGAATGTCATCATGTTTGTGTGCTTCGAACAGCTGAAGA
AGGAATTGATGAAGTCAAGGCAGACGGTGGATTGTACCACATAA

>*Microtus agrestis*
ATGGTGAGCCCAACAACTTCCGAAGTGCACCCCACCATGGGGGTCAAGATCTTCTCAGCTGGCATATCCGCCTGCCTGGCAGATATCATCACCTTCCCA
CTGGACACAGCCAAAGTCCGGCTTCAGATCCAAGGTGAAGGCCAGACCTCCACTACCATTAGGTATAAAGGTGTCCTGGGAACCATCACCACCCTGG
CAAAAACAGAAGGGTGGCCGAAACTGTACAGCGGTCTGCCTGCTGGCATTCAGAGGCAAATCAGCTTCGCCTCACTCAGGATCGGTCTCTATGATACT

GTCCAAGAGTACTTCTCTTCAGGGAAAGAAACGCCTCCCACTTTGGGAAACAGGATCTCAGCTGGCTTATTGACTGGTGGTGTGGCAGTATTCATCGG
ACAACCTACCGAGGTCGTGAAAGTCAGACTCCAAGCACAGAGTCACTTACACGGGATCAAACCCCGCTACACAGGGACCTACAATGCTTACAGAATT
ATAGCCACAACAGAAAGCTTGTCAACCCTCTGGAAAGGGACGACCCCTAATCTGATGAGAAATGTCATTATCAATTGTACAGAGCTGGTAACATATGA
CCTCATGAAGGGGGCCCTTGTGAACAACCAAATACTGGCAGACGACGTGCCCTGCCATTTACTGTCAGCTCTTGTGGCTGGGTTTTGCACCACCTTCC
TGGCCTCTCCGGCGGATGTGGTAAAAACAAGATTCATCAACTCTCTACCAGGACAGTACCCGAGTGTACCCAGCTGTGCAATGACCATGCTCACCAAG
GAAGGACCAACAGCTTTCTTCAAAGGGTTTGTGCCTTCTTTCCTGCGACTGGCGTCCTGGAATGTCATCATGTTTGTGTGCTTCGAACAGCTGAAGAA
GGAACTGATGAAGTCCAGGCAGACGGTGGACTGCACCACATAA

>*Neotoma lepida*
ATGGTGAGCCCGACAACTTCCGAAGTGCCTCCTACCATGGGNGTCAAGATCTTCTCAGCTGGCGTGGCCGCCTGCCTGGCAGATATCATCACCTTCCC
GCTGGACACAGCCAAAGTCCGGCTTCAGATCCAAGGTGAGGGCCAGACCTCCAGTGTTATTAGGTATAAAGGTGTCCTGGGGACCATCACCACTCTG
GCAAAAACAGAAGGGTTGCCGAAACTGTACAGCGGTCTGCCTGCTGGCATTCAGAGGCAAATCAGCTTTGCCTCACTCAGGATTGGCCTCTACGATA
CTGTCCAAGAGTACTTCTCTTCAGGGAAAGAAACACCTCCCACTTTGGGAAACAGGATCTCAGCTGGCTTAATGACAGGAGGTGTGGCAGTATTCATC
GGGCAACCTACAGAGGTTGTGAAAGTCAGACTCCAAGCACAGAGCCACCTACATGGGATCAAACCCCGCTACACTGGGACCTACAATGCTTACAGAA
TTATAGCTACAACAGAAAGCTTGTCAACACTCTGGAAAGGGACAACCCCTAATCTGATGAGGAATATAATCATCAATTGTACAGAGCTGGTGACATATG
ATCTCATGAAGGGAGCCCTTGTGAACAACCAAATACTGGCAGATGATGTCCCATGCCACTTACTGTCAGCTCTTGTCGCCGGATTTTGCACCACACTCC
TGTCCTCTCCAGTGGATGTGGTAAAAACAAGATTCATCAACTCTCTACCAGGACAGTACCCAAGTGTACCCAGCTGTGCAATGACCATGTTCACCAAG
GAAGGACCAACAGCTTTTCTCAAAGGGTTTGTGCCTTCTTTTCTGCGACTCGCATCTTGGAATGTCATCATGTTTGTGTGCTTTGAGCAGCTGAAAAAA
GAATTGATGAAGTCAAGGCAGACGGTGGACTGCGCCACATAA

>*Microtus fortis*
ATGGTGAGCCCGACAACTTCCGAAGTGCACCCCACCATGGGGGTCAAGATCTTTTCAGCTGGCATATCCGCCTGCCTTGCAGATATCATCACCTTCCCA
CTGGACACAGCCAAAGTCCGGCTTCAGATCCAAGGTGAAGGCCAGACCTCCACTACCATTAGGTATAAAGGTGTCCTGGGAACCATCACCACCCTGG
CAAAAACAGAAGGGTGGCCGAAACtgtacagtggtctgcctgctgGCATTCAGAGGCAAATCAGCTTCGCCTCACTCAGGATCGGTCTCTATGATACTGTCCAAGA
GTACTTCTCTTCAGGGAAAGAAACGCCTCCCACTTTGGGAAACAGGATCTCAGCTGGCTTATTGACTGGAGGTGTGGCAGTATTCATCGGACAACCTA
CTGAGGTCGTGAAAGTCAGACTCCAAGCACAGAGTCACTTACACGGGATCAAACCCCGCTACACAGGGACCTACAATGCTTACAGAATTATAGCCAC

AACAGAAAGCTTGTCAACTCTCTGGAAAGGGACGACCCCTAATCTGATGAGAAACGTCATTATCAATTGTACAGAGCTGGTAACATATGACCTCATGA
AGGGGGCCCTTGTGAACAACCAAATACTGGCAGACGACGTGCCCTGCCATTTACTGTCAGCTCTTGTGGCCGGGTTTTGCACCACCTTCCTGGCCTCT
CCAGCGGATGTGGTAAAAACAAGATTCATCAACTCTCTACCTGGACAGTACCCGAGTGTACCCAGCTGTGCAATGACCATGCTCACCAAGGAAGGAC
CAACGGCTTTCTTCAAAGGGTTTGTGCCTTCTTTCCTGCGACTGGCGTCCTGGAATGTCATCATGTTTGTGTGCTTCGAACAGCTGAAGAAGGAACTG
ACGAAGTCCAGGCAGACGGTGGACTGCACCACATAA

>*Dicrostonyx groenlandicus*
ATGGTGAGCTTGACAACTTCCGAAGTGCACCCCACCATGGGGGTCAAGACCTTCTCAGCTGGCATATCCGCCTGCCTGGCAGATATCATCACCTTCCCA
CTGGACACAGCCAAAGTCCGGCTTCAGATACAAGGTGAAGGCCAGACCTCCAGTACCATTAGGTATAAAGGTGTCCTGGGAACCATCACCACCCTGG
CAAAAACAGAAGGGTGGCCGAAACTGTACAGCGGTCTGCCTGCTGGCATTCAGAGGCAAATCAGCTTCGCCTCACTCAGGATTGGTCTCTATGATACT
GTCCAAGAGTACTTCTCTTCAGGAAAAGAAACACCTCCCACTTTGGGAAACAGGATCTCAGCCGGCTTAATGACTGGAGGTGTGGCAGTGTTCATCG
GGCAACCTACCGAGGTCGTGAAAGTCAGACTCCAAGCACAGAGCCACTTACATGGGATCAAACCCCGCTACACAGGGACCTACAATGCTTACAGAAT
TATAGCCACAACAGAAAGCTTTTCAACACTCTGGAAAGGGACGACCCCTAATCTGATGAGAAACGTCATTATCAATCGTACAGAGCTGGTAACATATG
ACCTCATGAAGGGGGCCCTTGTGAACAACCAAATACTGGCAGACGACGTGCCATGCCATTTACTGTCAGCTCTTGTTGCCGGGTTTTGCACCACCTTC
CTGGCCTCTCCGGCGGATGTGGTAAAAACAAGATTCATCAACTCTCTACCAGGACAGTACCCGAGTGTACCCAGCTGTGCAATGACCATGCTCACCAA
GGAAGGACCAACGGCTTTTTTCAAAGGGTTTGTGCCTTCTTTCCTGCGACTGGCGTCCTGGAATGTCATCATGTTTGTGTGCTTTGAACAGTTGAAGA
AAGAATTGATGAAGTCAAGACAGACGATGGACTGCACCACATAA

>*Phodopus sungorus*
ATGGTGAGCCAGACAACTTCCGAAGTGCAACCCACCATGGGGGTCAAGATCTTCTCAGCCGGCGTGGCGGCCTGCCTGGCAGATATCATCACCTTCCC
GCTGGACACGGCCAAAGTCCGGCTTCAGATCCAAGGTGAAGGCCAGACCTCCAGTACCATTAGGTATAAAGGTGTCCTTGGGACCATCACCACCCTG
GCAAAAACAGAAGGGTTGCCGAAACTGTACAGTGGTCTGCCCGCTGGTATCCAGAGACAAATCAGCTTTGCCTCACTCAGGATTGGCCTCTACGATAC
TGTCCAAGAGTACTTCTCTTCGGGGAAAGAAACTCCTCCCACTTTGGTAAACAGGATCTCAGCTGGCTTAATGACTGGAGGTGTGGCAGTATTCATTG
GGCAACCTACTGAGGTTGTGAAAGTCAGACTCCAAGCACAGAGCCACCTACACGGGATCAAACCCCGCTACACGGGTACCTACAATGCTTACAGAAT
TATAGCCACAACAGAAAGCCTGTCAACACTCTGGAAAGGGACAACCCCTAATCTGTTGAGGAATGTCATCATCAACTGTACAGAGCTGGTAACATATG
ACCTCATGAAGGGGGCCCTTGTGAACAACCAAATATTGGCAGATGACGTGCCATGCCACTTACTGTCAGCTCTTGTCGCCGGCTTTTGCACCACATTCC

TGGCCTCTCCAGCAGATGTGGTAAAAACAAGATTCATCAACTCTCTACCAGGACAGTACCCAAGTGTACCCAGCTGCGCAATGACCATGTTCACCAAG
GAAGGACCAACAGCTTTTTTCAAAGGGTTTGTGCCTTCTTTCCTGCGACTCGCATCCTGGAATGTCATCATGTTTGTGTGCTTTGAACAGCTGAAGAA
AGAGTTGATGAAGTCGAGGCAGACGGTGGACTGCACCACATAA

>*Marmota himalayana*

ATGGTGAGCCCCACAGCCTCCGACGTGCACCCGACCATGGGCATCAAGATCGTCTCAGCCGGAGTGTCAGCCTGCTTGGCGGATGTGATCACCTTCCC
GCTGGACACCGCCAAAGTCCGGCTACAGATCCAAGGCGAATTCCCAGTCTCCAGTGGTATTAAGTATAAAGGTGTCCTGGGAACAATCACCACCCTGG
CAAAAACCGAAGGGCCCATGAAACTGTACAGTGGGTTGCCTGCGGGATTGCAAAGGCAAATAAGCTTCGCCTCTCTTAGGATCGGCCTCTATGATTCT
GTCCAGGAGTTCTTCACCTCAGGGAATGAAACAACACCCAGTTTGGGAAGCAAAATCTCAGCCGGACTCACAACTGGAGGAGTGGCAGTGTTCATTG
GGCAGCCCACCGAGGTCGTGAAGGTCAGGCTCCAAGCACAGAGCCACCTACATGGGCTCAAACCTCGCTACACTGGGACCTACAATGCGTACAGAAT
TATAGCAACAACAGAGAGCTTCAGGAGTCTTTGGAAAGGGACTACTCCTAATCTGTTGAGAAATGTCATCATCAATTGCACAGAGTTGGTAACATATG
ACCTGATGAAGGGGGCTCTTGTGAGAAACAAAATCCTAGCAGATGATGTCCCCTGCCACCTGCTGTCCGCTTTCGTCGCTGGATTTTGCACCACACTT
CTGTCCTCGCCGGCTGATGTGGTGAAGACCAGATTTATTAACTCTCCGCCAGGGCAGTATACCAGCGTGCCCAGATGTGCAATGACAATGCTCCACCA
GGAAGGGCCGTCGGCGTTTTTCAAAGGATTTGTACCTTCCTTCCTGCGACTGGCATCCTGGAACGTCATTATGTTCGTGAGCTTTGAACAGCTGAAAC
GAGAATTGATGAAGTCCAGGCAGACAGTGGACTGCGCCACGTGA
